# Changes in longevity inequality by education among OECD countries before the COVID-19 pandemic

**DOI:** 10.1186/s12889-023-16492-z

**Published:** 2023-08-28

**Authors:** Christopher Lübker, Fabrice Murtin

**Affiliations:** 1 University of York Centre for Health Economics, York, UK; 2 OECD, 2 Rue André Pascal, 75016 Paris, France

**Keywords:** Longevity, Health inequality, Life expectancy, Socio-economic gradient

## Abstract

**Background:**

Disparities in life expectancy between socioeconomic groups are one of the main challenges for health policy, and their reduction over time is an important policy objective.

**Methods:**

Observational study using routinely registered data on mortality around 2011 and 2016 by sex, age, educational attainment level, and cause of death in 13 member countries of the Organization for Economic Cooperation and Development (OECD). The main outcome measures are life expectancy by education at the ages of 25 and 65 in 2011 and 2016.

**Results:**

Between 2011 and 2016, the life expectancy gap has increased by 0·2 years among men and 0·3 years among women from 13 available countries. The United States recorded one the largest increases in the absolute life expectancy gap, 1·3 years for women and 1·1 years for men respectively.

**Conclusion:**

Inequality in longevity has increased in over half of the countries surveyed and starkly so in the United States in a context of deteriorating health.

**Trial registration:**

Not applicable.

**Supplementary Information:**

The online version contains supplementary material available at 10.1186/s12889-023-16492-z.

## Background

People with lower educational attainment tend to live shorter lives in worse health [1, 2]. Longevity is an essential component of well-being and therefore a policy concern in its own right. Moreover, the economic losses associated with premature and avoidable deaths have been estimated to cost 1.4% of gross domestic product annually in the European Union (EU) [3].

International organisations and their member countries have recognised the urgent need to address health inequalities [4]. Cross-country studies monitoring (socioeconomic) health inequalities allow policymakers and researchers to track progress towards inequality reduction targets and inform evidence-based policy design. However, international studies encounter data limitations and methodological challenges, which have made comprehensive studies rare, even in high-income countries [5–7] and especially outside Europe [8–10]. This paper updates the analysis of educational inequalities in longevity by Murtin and colleagues between and within 21 high-income countries around 2016, drawing on data from diverse regions (North America, Oceania, East Asia, and Europe) [11, 12]. The obtained data are useful to document inequalities related to specific causes of death and they have served as an input to the European Cancer Inequalities Registry, which is a flagship initiative of the Europe’s Beating Cancer Plan. Moreover, as a main contribution to the literature, this paper is able to document the changes of inequality in longevity across population groups within 13 countries observed in both 2011 and 2016.

## Methods

### Data

The Organisation for Economic Co-operation and Development (OECD) collected country-level mortality and person-year population exposure from 21 member countries in 2013–19 to calculate longevity by age, sex and education. Participation to the data collection was non-mandatory and relied on pro-bono inputs from national health specialists. While a significant number of countries declined to participate, the data collection resulted in a total population exposure of over 1·4 billion person-years. Of the 21 countries, nine use data from death certificates linked to educational qualification registers while data from the remaining countries use unlinked data. Age is recorded in single-year increments from age 25 to age 120, although some countries apply cut-offs beyond age 85, or report age in 5-year groups (see Annex A.1 in Supplementary Information).

Education level is categorised using the 2011 International Standard Classification of Education (ISCED-2011) into low (lower secondary education and below, ISCED 0–2), medium (upper secondary, ISCED 3–4), and high education (higher than upper-secondary, ISCED 5–8) [13]. Missing education was recorded for eight countries, the treatment of which is discussed in Supplementary Information Annex A, where the distributions of education for each country-source are also available [14].

Cause of death data were available for 14 countries around 2016 (see Table A.1 in Supplementary Information), and were categorised using the 10th revision of the International Classification of Diseases (ICD-10) [15]. To focus on the most common causes of death globally, this study groups deaths according to circulatory system diseases (ICD-10 Chapter IX: I00-I99), neoplasms (ICD-10 Chapter II: C00-D48), external causes (ICD-10 Chapter XX: V01-Y98), and all other causes.

### Calculating educational inequalities in longevity

Life expectancy at age 25 and 65 is calculated for each gender and education group using the Chiang abridged life table method [16]. The gap in life expectancy between high and low education groups is the primary outcome measure of inequality in longevity. As complementary measures of inequalities in longevity, we consider absolute and relative measures of inequality in age-standardised mortality rates, i.e., the absolute difference in, and the ratio of, age-standardised mortality rates between high and low education groups, in the population aged 25 to 89. The contribution of each cause-of-death categories was grouped to approximate the working- and retirement-age populations, ages 25 to 64 and 65 to 89, respectively.

In Annex B, we also present the results of the slope index of inequality (SII) and its relative transformation, the relative index of inequality (RII) [17]. These measures take into account cross-country differences in the size of the three education groups and are immune to composition effects that would distort the inequality index if a specific educational group were over or under-represented relative to other countries. In this study, the SII (RII) describes the linear prediction of the absolute (relative) difference in age-standardised mortality rates between the high and low education groups. Furthermore, in Annex B, we report the contribution of between- and within-group differences to educational inequalities in age-at-death, using the Theil Index [6]. Methods for these analyses are detailed in Annex A.

## Results

This Section first examines results pertaining to 21 countries observed around 2016, before highlighting the changes in the educational inequalities in life expectancy among 13 countries between 2011 and 2016.

**Fig. 1:**
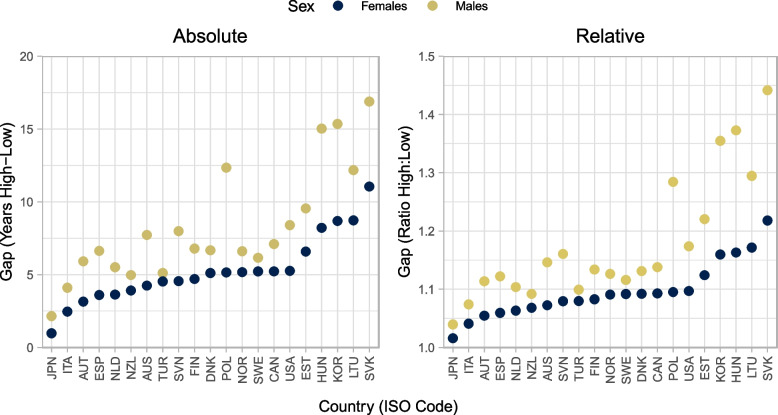
Absolute and relative life expectancy gaps between high and low education groups at age 25 by country and sex around 2016. Note: Countries are reported in International Organization for Standardization (ISO) three-letter codes. Education is classified according to the 2011 International Standard Classification of Education (ISCED-2011) into low (lower secondary education and below, ISCED 0-2), medium (upper-secondary, ISCED 3-4), and high education (higher than upper-secondary, ISCED 5-8)

Figure 1 shows the absolute and relative gaps in life expectancy between high and low education groups. The average absolute gap in female and male life expectancy at age 25 is 5·3 and 8·3 years, respectively. Absolute gaps in male life expectancy are greater than 10 years in Poland, Lithuania, Hungary, Korea, and the Slovak Republic, and greater than 15 years in the latter three countries. Absolute gaps in female life expectancy are less than four years in Austria, Spain, Italy, Japan, Netherlands, New Zealand, and Slovenia. In USA, the absolute gap in life expectancy between high and low education groups is 5.3 years for women and 8.4 years for men (see full results in Table B.2 Annex B).

Relative gaps tell much the same story as absolute gaps. Life expectancy at 25 in the high education group is approximately 10% higher than the low education group among women and 18% higher than the low education group among men. In Poland, Lithuania, Korea, Hungary, and the Slovak Republic, men in the high education group have life expectancies over 25% longer than men in the low education group. In Australia, Austria, Spain, Italy, Japan Netherlands and New Zealand, women in the high education group have life expectancies less than 7.5% longer than women in the low education group.

**Fig. 2:**
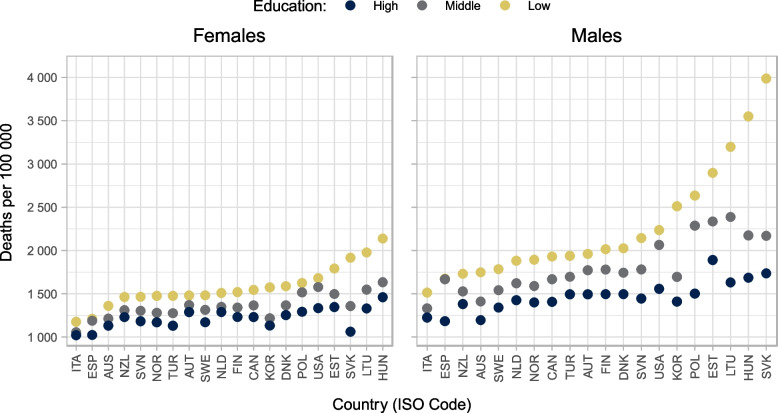
Age-standardised mortality rates by country, sex, and education around 2016. Note: Countries are reported in International Organization for Standardization (ISO) three-letter codes. Education is classified according to the 2011 International Standard Classification of Education (ISCED-2011) into low (lower secondary education and below, ISCED 0-2), medium (upper-secondary, ISCED 3-4), and high education (higher than upper-secondary, ISCED 5-8). Mortality rates are standardised using the OECD 2010 standard population

Figure 2 shows the age-standardised mortality rates by education level for women and men, in deaths per 100 000 person-years (full results available in Supplementary Information Annex B). Women in low, middle, and high education groups have standardised mortality rates of approximately 1 570, 1 350, and 1 220 deaths per 100 000, respectively, while men in low, middle, and high education groups have standardised mortality rates of approximately 2 270, 1 820, and 1 480 deaths per 100 000. We observe higher standardised mortality rates than the sample average across all education groups for both women and men in Hungary, Lithuania, Poland, and the United States. Conversely, Australia, Denmark, Italy, Sweden, and Türkiye have standardised mortality rates below the sample average for all education groups for women and men (see full results in Table B.3 Annex B).

**Fig. 3:**
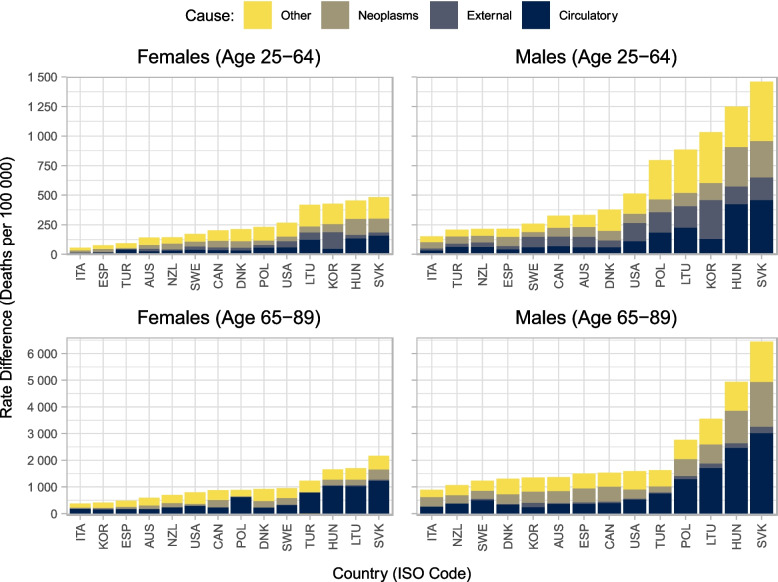
Decomposition of contribution to rate differences in age-standardised mortality rates by country, sex, age-group, and cause of death around 2016. Note: Countries are reported in International Organization for Standardization (ISO) three-letter codes. Mortality rates are standardised using the OECD 2010 standard population. Circulatory system diseases (Chapter IX): I00-I99. Neoplasms (Chapter II): C00-D48. External causes (Chapter XX): V01-Y98. Other diseases: All other causes

Figure 3 shows contributions to the total rate differences in age-standardised mortality rate between low and high education groups by cause of death. The relative contribution of circulatory disease to total rate differences in standardised mortality rates is higher among 65- to 89-year-olds than among 25- to 64-year-olds for men and women across all countries. Conversely, the proportion of external disease contribution to rate differences in standardised mortality rates is greater among 25- to 64-year-olds than among 65- to 89-year-olds, and greater for men than for women. Among women and men ages 25 to 64, deaths from causes other than circulatory disease, neoplasm, or external causes, account for over 47% and 35% of the risk difference in age-standardised mortality rates, respectively. Among women and men aged 65–89, deaths from circulatory diseases account for 49% and 41% of the risk differences in age-standardised mortality rates, respectively. The contribution to the total rate difference due to deaths from external causes is particularly high in Sweden, Korea, and the United States, which may be partly explained by deaths of despair [18]. Relative contributions are presented in Supplementary Information Annex B.

Table 1 shows the differences between inequality measurements from the 2011 analysis provided by Murtin and colleagues compared to the 2016 update presented in this paper [11, 12]. Data treatments and sources are identical across the two studies. Due to methodological and data limitations in the 2011 analysis, data from Canada and the Slovak Republic are excluded from this comparison (further details are provided in Annex A). In the remaining sample of 13 countries, absolute gaps in life expectancy by educational level have increased by 0.4 years for women and 0.5 years for men over 4.9 years on average. This increase is primarily driven by Australia, Hungary, Portugal, and the United States. The United States recorded the largest increase in the absolute gap in life expectancy after Hungary. Overall, inequality in longevity has increased in over half the countries surveyed. Some countries have experienced reductions in the life expectancy gap, including Denmark, Estonia, Poland, and Slovenia. These trends are also reflected in the relative life expectancy gap.

**Table 1:** Change in life expectancy inequalities by education from data around 2016 and 2011

Country	Year Average (2011 / 2016 Analysis)			Δ Absolute LE Gap		Δ Relative LE Gap	
				**Females**	**Males**	**Females**	**Males**
AUS	2011	/	2016	0.6	1.1	0.009	0.020
AUT	2012	/	2017	0.1	-0.5	0.002	-0.011
DNK	2011	/	2015	0.0	-0.2	-0.003	-0.007
FIN	2010	/	2015.5	-0.1	-0.8	-0.002	-0.021
HUN	2011.5	/	2017.5	2.5	1.1	0.051	0.023
ITA	2012	/	2014.5	0.5	0.3	0.007	0.004
NOR	2011	/	2015.5	0.4	-0.2	0.005	-0.007
NZL	2006	/	2015.5	-0.5	0.3	-0.010	0.003
POL	2011.5	/	2016.5	-1.0	-0.3	-0.021	-0.013
SVN	2011.5	/	2015.5	-0.1	-0.3	-0.002	-0.009
SWE	2012.5	/	2016.5	0.2	0.3	0.004	0.005
TUR	2013	/	2014.5	0.6	1.0	0.009	0.018
USA	2011.5	/	2018.5	1.3	1.1	0.025	0.025
**Average**	**4.9 years**			**0.3**	**0.2**	**0.006**	**0.002**

*Note*: Data were provided from Murtin and colleagues [11, 12]. Countries are reported in International Organization for Standardization (ISO) three-letter codes. Gaps are between high and low education groups according to the 2011 International Standard Classification of Education (ISCED-2011): low (lower secondary education and below, ISCED 0-2) and high (higher than upper-secondary, ISCED 5-8). *Abbreviations*: *LE* life expectancy

## Discussion

The key findings of the paper are the following. First, absolute (relative) gaps in life expectancy at age 25 between high and low education groups around 2016 were five and eight years 10% and 18%) for women and men, respectively. Absolute (relative) gaps were lowest in Japan, at one year for women (2%) and two years for men (4%), and highest in the Slovak Republic, at 11 years for men (22%) and 17 years (42%), for women and men, respectively. Second, circulatory diseases remain the main contributor to educational inequalities in age-standardised morality after age 65. Among women and men ages 65 to 89, deaths from circulatory diseases account for 49% and 41% of the differences in age-standardised mortality rates, respectively. For women and men aged 25–64, deaths from causes other than circulatory disease, neoplasm, or external causes account for over 47% and 35% of the difference in age-standardised mortality rates on average, respectively. Finally, comparing identical country-sources to the previous analysis centred on 2011, absolute gaps in life expectancy at age 25 have increased by 0·3 years for women and 0·2 years for men. Absolute gaps in life expectancy have grown by more than one year in Australia (men only), Hungary, the Republic of Türkiye (men only), and the United States.

Patterns of mortality inequality by education within Europe have been reported before, but they are mostly available for European OECD countries [7]. These studies noted large and increasing absolute and relative inequalities in Eastern European countries and low inequalities in Italy and Spain. Recent studies in Australia [19, 20] and Japan [21] report results similar to those in this paper. Differences between Australian estimates may be due to the maximum age used in the life table calculation of life expectancy. Other estimates confirm the lower life expectancy found in international studies with fewer comparators which disproportionately affects those with lower educational attainment, particularly in the US [8, 22–24]. In the US health inequalities are increasing while life expectancy is stagnating; this may be attributed to rising midlife mortality, concentrated among groups with lower educational attainment [22–27]. A key component of the rise in midlife mortality is deaths of despair – including deaths from suicide, drug overdose, and liver cirrhosis [28] – which contribute 0·7 years to the female life expectancy gap at age 25 between high and low education groups in the US and 1·5 years to the male life expectancy gap [18].

We found that deaths from other causes than cardiovascular disease, cancer, and external causes accounted for most of the disparities in mortality between education groups before age 65, while cardiovascular disease accounts for a large share of mortality inequalities after age 65. The heterogeneous category of “other diseases” includes diabetes, Chronic Obstructive Pulmonary Disease, infectious diseases such as pneumonia, tuberculosis and HIV/AIDS, alcohol-related health problems and opioid-involved poisoning. Some of these diseases may reflect disparities in access or quality of medical care, whereas others may reflect the effect of disparities in smoking, excessive alcohol consumption, obesity or mental health.

This paper is subject to limitations. Data quality varies between country-sources; unlinked and self-reported data may be subject to misreporting [29]. Furthermore, subgrouping data by age, sex, education, and cause of death may lead to small sample sizes and volatile mortality rate estimates. We grouped ages into five-year groups and pooled data across available years to minimise this problem. Where observation counts are especially small due to subgrouping, some data were censored to protect anonymity of the deceased, which may influence results. We have made simplifying assumptions in the absence of data to inform our data treatment, including (a) mortality rates generally rise log-linearly at all successive ages from age 30 and (b) education group mortality rates never cross over at ages beyond 85. We have endeavoured to treat and analyse data to maximise comparability between countries, however some differences remain. Countries do not all have data available for the same years, hence the average year of the pooled data may differ. The biggest difference is between Japan (average 2010) and the United States (average mid-2018). Assuming within-country variations across years are smaller than between-country variations, country ranks will hold across different inequality measures.

Routine updates are required to monitor international educational inequalities in longevity, tracking progress towards reducing inequalities and informing evidence-based policy-making and target-setting. Where possible, these data should transition towards a linked collection methodology, whereby the death certificate is directly linked to administrative data to ensure the reliability of the data. Future research could expand country-coverage and assess longevity inequality by other socioeconomic characteristics, such as ethnicity or race. Expanding research on stratifying variables and country inclusion is subject to subject to data availability and quality, which has been the reason for their exclusion in this analysis.

## Conclusion

This study presents new harmonized data on mortality and population by age, sex, education, and causes of death for 21 high-income OECD countries around 2016, and it calculates the changes of educational inequality in longevity between 2011 and 2016 among 13 countries. The results reveal that both absolute and relative gaps in life expectancy by education at age 25 have increased on average across countries between 2011 and 2016. The study also points to an unfavourable position of the United States that record one the largest increase in the absolute gap in life expectancy between high and low education groups (1·3 and 1·1 years for women and men, respectively). These findings constitute a call to national health authorities and policy-makers to improve access to high-quality healthcare and implement public health interventions to reverse this trend.

### Supplementary Information

**Additional file 1:** **Annex A.** Data characteristics. **Annex B.** Additional results.

## Data Availability

The datasets used and/or analysed during the current study are available from the corresponding author on reasonable request.
