# Supplementary material for: Changes in longevity inequality by education among OECD countries before the COVID-19 pandemic
Source: BMC Public Health. 2023 Aug 28;23:1646. doi: 10.1186/s12889-023-16492-z (PMC10464106; doi:10.1186/s12889-023-16492-z)
Supplement: Supplementary file 1 — Additional file 1: Annex A. Data characteristics. Annex B. Additional results. [file 12889_2023_16492_MOESM1_ESM.docx]

CHANGES IN LONGEVITY INEQUALITY BY EDUCATION AMONG OECD COUNTRIES BEFORE THE COVID-19 PANDEMIC: ONLINE APPENDIX

ANNEX A. DATA CHARACTERISTICS

### A.1. Country-Source Assessments

The data sources differ in their collection methodologies. For the purposes of this study, the key methodological difference relates to linking death certificates to educational qualification data. In linked data, death certificates are directly linked to administrative data containing educational qualifications of the deceased, either directly (via a social security number) or indirectly (via probabilistic linkage on individual characteristics, such as date of birth and address). In unlinked data, educational attainment is reported at the time of death by relatives or public officials, which may lead to misreporting bias.^29^

This study is based on an analysis described in Murtin and Lübker,^18^ where overall data quality is assessed on a five-star scale for each country-source, five stars indicating the highest quality and one star the lowest quality data available for this analysis. For all sources, one star was subtracted for each of the following cases: (1) unlinked mortality data, (2) cause of death unavailable, (3) use of rolling population estimates, and (4) use of asymmetrical rolling population estimates, where mortality and population exposure data do not refer to the same year. For this study, we use only the highest quality data with three stars or more as primary sources for analyses (Table A.1) The combination of sources results in 21 countries covered by primary sources. For each country-source, we pool available data between 2013-19 to maximise sample sizes and smooth random variation in mortality rates.

Table A.1. Data overview

|  |  |  |  |  |  |  |  |  |  |  |
| --- | --- | --- | --- | --- | --- | --- | --- | --- | --- | --- |
|  |  |  |  |  |  |  |  |  |  |  |
| **Country** | **Source** | **Data Quality** | **Primary  Source** | **Death  Registers** | **Population  Exposure** | **Analysis Period** | **Age Range** | **Linked** | **Cause of Death** | **Notes** |
| AUS | OECD | ***** | Yes | 2016-17 | 2016 | 2016 | 25-100+ | Yes | Yes | 1 |
| AUT | OECD | **** | Yes | 2016-17 | 2016 | 2017 | 25-95+ | Yes | No |  |
| CAN | OECD | **** | Yes | 2012-16 | 2012-16 | 2013-16 | 25-90+ | Yes | Yes | 1,3 |
| DNK | OECD | **** | Yes | 2013-17 | 2013-17 | 2014-17 | 25-120 | No | Yes |  |
| ESP | OECD | **** | Yes | 2016-18 | 2016-18 | 2016-18 | 25-100 | Yes | Yes |  |
| EST | Eurostat | *** | Yes | 2011-18 | 2015-20 | 2015-18 | 25-99 | No | No |  |
| FIN | Eurostat | *** | Yes | 2011-18 | 2011-20 | 2013-18 | 25-99 | No | No |  |
| HUN | OECD | **** | Yes | 2011-19 | 2016 | 2016-19 | 25-120 | No | Yes |  |
| ITA | OECD | ***** | Yes | 2012-16 | 2012-16 | 2013-16 | 25-120 | Yes | Yes |  |
| JPN | OECD | *** | Yes |  |  | 2010; 13-15 | 25; 65 | Yes | No | 4 |
| KOR | OECD | *** | Yes | 2015-18 | 2015 | 2015-18 | 25-84 | No | Yes |  |
| LTU | OECD | **** | Yes | 2015-19 | 2015-19 | 2015-19 | 25-120 | Yes | Yes |  |
| NLD | OECD | *** | Yes |  |  | 2015-2018 | 25-99+ | ? | No | 5 |
| NOR | Eurostat | *** | Yes | 2011-18 | 2011-20 | 2013-18 | 25-99 | No | No |  |
| NZL | OECD | ***** | Yes | 2013-18 | 2013-18 | 2013-18 | 25-95+ | Yes | Yes | 1 |
| POL | OECD | **** | Yes | 2014-19 | 2011 | 2014-19 | 25-120 | No | Yes |  |
| SVK | OECD | **** | Yes | 2015-18 | 2015-18 | 2015-18 | 25-120 | No | Yes |  |
| SVN | Eurostat | *** | Yes | 2011-18 | 2011-12;14-20 | 2015-18 | 25-99 | No | No |  |
| SWE | OECD | ***** | Yes | 2015-18 | 2015-18 | 2015-18 | 25-120 | Yes | Yes |  |
| TUR | OECD | **** | Yes | 2014-15 | 2014-15 | 2014-15 | 25-100+ | No | Yes |  |
| USA | OECD | **** | Yes | 2018-2019 | 2018-2019 | 2018-2019 | 25-85+ | No | Yes | 1 |

Note: (1) Data provided in 5-year age-groups; (2) Exposure and mortality data are asymmetrical. We assume exposure values are equal to those in the first available year with mortality data; (3) Mortality data from CanCHEC are adjusted to reflect the structure of the Canadian population; (4) Raw exposure and mortality data not provided and probabilistic matching performed to link data as reported by Kasajima and colleaguges;^21^ (5) Raw exposure and mortality data were not provided, 1-year mortality probabilities converted to rates. Countries are reported in International Organization for Standardization (ISO) three-letter codes

There is some heterogeneity in data collection methodologies and standardisations. Raw exposure and deaths data were not collected for Japan, where life expectancy estimates at 25 (for 2000-15) and 65 (for 2010) were provided directly for each sex-education group by Kasajima and colleagues, with probabilistic matching-based linkages.^21^ Japanese estimates are included in this paper from 2013-15 for life expectancy at 25, and in 2010 for life expectancy at 65. Data from the Netherlands were provided in single-year age group mortality probabilities from birth to age 99 by sex and education, averaged across the period 2015-18. These one-year probabilities are transformed to rates using standard probability-rate conversion methodologies. Finally, the Canadian data provided under-reported mortality rates, since they exclude individuals living in institutions, collective housing, and the homeless.^18^ We applied corresponding average age-sex specific correction factors from 2011-16 to reflect the Canadian population, assuming the same correction factor for each education group. The 2011 analysis did not use and adjustment factor to align CanCHEC data with the general Canadian population, which may explain a large proportion of differences in results between the analysis of Murtin and colleagues on 2011 data.^11,12^ For the US, we applied the correction proposed by Rostron and colleagues described later in this Annex.^14^

To maximise country coverage, unlinked data from the European Union’s (EU) Eurostat databases were used to include 4 additional countries (Estonia, Finland, Norway, and Slovenia). Mortality and exposure data are available for 1-year age groups by country, education. Beyond age 75, Eurostat data are censored and do not differentiate education-specific data, referring instead to the population-level data trends by sex. Our data treatment corrects for the latter issue.

### A.2. Data treatments

Eight countries report missing education data. According to Eurostat guidance and the methodology of Murtin and colleagues, missing education data are proportionally assigned according to the observed exposure in low, middle, and high education groups, respectively, for each country-age-sex group.^11,12^ Alternatively, all missing education data may be assigned to the low education category, on the assumption that missing education data are more likely to be attributable to low education individuals. We explore the impact of imputing all missing data to the low education group in a robustness check shown below.

In some instances, country-age-sex-education specific mortality rates were corrected to: (1) predict missing data points beyond country-specific age cut-offs; (2) smooth the random variation mortality rates, which may result in volatile trends between age-groups; and/or (3) prevent implausible cross-overs between education group mortality trends. Correction 1 ensures comparability across countries. Correction 2 standardises results according to Gompertz’s law, predicting that log mortality rates rise linearly from age 30 in each successive age group.^18^ Correction 3 is imposed sparingly when small samples may spuriously lead to lower mortality rates for lower education groups than higher education groups, a pattern most commonly occurring above age 85. In such cases, we impose a simple convergence rule, whereby the mortality rate of the low education group cannot fall below that of the middle education group, and the mortality rate of the middle education group cannot fall below that of the high education group. These corrections are applied for the sake of consistency and have small impacts on longevity estimates, since they occur at high age. We correct mortality rates by extrapolating log-linear trends:

$$\log m_{i,j,t}= a_{i,j}+b_{i,j}.t+\varepsilon_{i,j,t}$$

where m_i,j,t_ is the the mortality rate of sex-education-age group, *i*, *j*, and *t*, respectively. We use the five preceding five-year age-group data points to infer the predicted log mortality rates from a given age. Raw and predicted log mortality rate figures by age, sex, education, country, and source are available in Annex 2 and 3, respectively.

US death registers use two types of education classification, either grade-based or degree-based. After discussion with OECD experts on the US education system, grades and degrees were allocated to low, medium or high educational attainment categories according to the mapping described in Tables A2-A3. In addition, Rostron and colleagues propose a correction factor of the number of deaths by educational level recorded in death registers, which is used in this paper and reported in Table A4.^14^

Table A.2. Distribution of educational attainment by country, source, and age for women

| **Country** | **Source** | **Total Person-Years** | **Age 25-44** | | | | | **Age 45-64** | | | | | **Age 65-84** | | | | | **Age 85+** | | | | |
| --- | --- | --- | --- | --- | --- | --- | --- | --- | --- | --- | --- | --- | --- | --- | --- | --- | --- | --- | --- | --- | --- | --- |
|  |  | **(1,000,000s)** | **Person-Years** | **Low** | **Middle** | **High** | **Missing** | **Person-Years** | **Low** | **Middle** | **High** | **Missing** | **Person-Years** | **Low** | **Middle** | **High** | **Missing** | **Person-Years** | **Low** | **Middle** | **High** | **Missing** |
|  |  |  | **(1000s)** | **(%)** | **(%)** | **(%)** | **(%)** | **(1000s)** | **(%)** | **(%)** | **(%)** | **(%)** | **(1000s)** | **(%)** | **(%)** | **(%)** | **(%)** | **(1000s)** | **(%)** | **(%)** | **(%)** | **(%)** |
| AUS | OECD | 8 | 3,413 | 11.8 | 49 | 29 | 10.2 | 2,913 | 22 | 47.1 | 20.7 | 10.2 | 1,538 | 30.8 | 41 | 13.7 | 14.4 | 179 | 35.6 | 31.9 | 8 | 24.5 |
| AUT | OECD | 3.1 | 1,185 | 16.6 | 50.6 | 32.8 |  | 1,251 | 14.1 | 57.9 | 28 |  | 629 | 22.1 | 55 | 22.9 |  | 70 | 31.6 | 46.8 | 21.6 |  |
| CAN | OECD | 42.8 | 13,828 | 10.7 | 38.9 | 50.4 |  | 18,970 | 14.8 | 41.3 | 43.9 |  | 9,120 | 25.8 | 38.6 | 35.5 |  | 928 | 40.5 | 33.9 | 25.6 |  |
| DNK | OECD | 9.7 | 3,580 | 19.1 | 43.3 | 34.6 | 3 | 3,767 | 23.4 | 47.5 | 27.1 | 2 | 2,197 | 31.3 | 43.7 | 22.9 | 2.1 | 192 | 44.1 | 30.2 | 16.5 | 9.2 |
| ESP | OECD | 50.8 | 19,686 | 42.4 | 22.3 | 35.4 |  | 19,601 | 48.7 | 21.8 | 29.5 |  | 10,082 | 70.5 | 12.4 | 17.1 |  | 1,446 | 83.8 | 6.9 | 9.3 |  |
| EST | Eurostat | 1.8 | 768 | 20.7 | 49.7 | 27.7 | 1.9 | 649 | 7.8 | 57.1 | 34.1 | 1.1 | 316 | 14.2 | 50.2 | 35.2 | 0.4 | 27 | 22 | 42.4 | 35.4 | 0.3 |
| FIN | Eurostat | 11.5 | 4,198 | 18.2 | 49.8 | 32 |  | 4,400 | 21.8 | 46.3 | 31.9 |  | 2,619 | 45.7 | 28.2 | 26.2 |  | 237 | 68.4 | 13.6 | 18 |  |
| HUN | OECD | 13.2 | 5,257 | 13.1 | 61.1 | 25.8 |  | 5,069 | 14.8 | 66.5 | 18.6 |  | 2,654 | 30.7 | 48.2 | 21.1 |  | 199 | 59.1 | 16.4 | 24.5 |  |
| ITA | OECD | 81.9 | 26,488 | 37.1 | 45.4 | 17.5 |  | 32,912 | 51.2 | 36 | 12.8 |  | 20,111 | 72.9 | 18.8 | 8.2 |  | 2,434 | 82.2 | 11 | 6.8 |  |
| JPN | OECD |  |  |  |  |  |  |  |  |  |  |  |  |  |  |  |  |  |  |  |  |  |
| KOR | OECD | 69.7 | 27,275 | 1.2 | 24.3 | 74.5 |  | 31,036 | 15.1 | 42.6 | 42.3 |  | 11,196 | 50.1 | 30 | 19.9 |  | 198 | 64.4 | 18.4 | 17.2 |  |
| LTU | OECD | 4.6 | 1,799 | 20.6 | 48.4 | 31 |  | 1,891 | 6.4 | 67.2 | 26.4 |  | 864 | 28.3 | 50.3 | 21.4 |  | 85 | 57.6 | 24.7 | 17.6 |  |
| NLD | OECD |  |  |  |  |  |  |  |  |  |  |  |  |  |  |  |  |  |  |  |  |  |
| NOR | Eurostat | 10.7 | 4,358 | 19 | 36.1 | 36.1 | 8.8 | 4,044 | 20.4 | 44.7 | 31.9 | 3 | 2,074 | 25 | 48.6 | 25.8 | 0.6 | 227 | 36.1 | 45 | 17.9 | 1 |
| NZL | OECD | 20.8 | 8,019 | 21 | 35.2 | 29.9 | 13.8 | 8,214 | 29.1 | 33 | 25.8 | 12.1 | 4,114 | 36.8 | 27.8 | 18.1 | 17.4 | 403 | 36.4 | 25.8 | 13.8 | 24 |
| POL | OECD | 75 | 31,450 | 7.9 | 60 | 24.2 | 7.9 | 30,592 | 13.6 | 69.7 | 12.4 | 4.3 | 12,024 | 34.3 | 49.5 | 14 | 2.1 | 896 | 58.3 | 26.1 | 13 | 2.6 |
| SVK | OECD | 7.7 | 3,505 | 6.6 | 67.5 | 25.9 |  | 2,848 | 7.2 | 75.1 | 17.7 |  | 1,212 | 11.9 | 70.9 | 17.2 |  | 87 | 20.3 | 63.4 | 16.2 |  |
| SVN | Eurostat | 3.1 | 1,206 | 9.9 | 63.7 | 26.5 |  | 1,210 | 18.8 | 63.4 | 17.7 |  | 592 | 25.9 | 57.5 | 16.6 |  | 49 | 32.1 | 49.2 | 18.6 |  |
| SWE | OECD | 14 | 5,351 | 11.5 | 50.8 | 34.2 | 3.5 | 4,988 | 16.1 | 58.2 | 24.6 | 1.1 | 3,287 | 32.8 | 46.1 | 19.8 | 1.2 | 361 | 48 | 36.2 | 13.9 | 2 |
| TUR | OECD | 45.6 | 24,380 | 44.5 | 29.6 | 24.5 | 1.5 | 15,743 | 65.3 | 19.2 | 14.3 | 1.2 | 5,202 | 82 | 7.4 | 8.7 | 1.9 | 313 | 86.7 | 5.2 | 6.1 | 2 |
| USA | OECD | 215.8 | 87,344 | 11.1 | 48.1 | 40.8 |  | 81,833 | 12.6 | 49 | 38.4 |  | 42,130 | 13.4 | 46.3 | 40.2 |  | 4,452 | 21.8 | 45.6 | 32.7 |  |
| **Average** | | **36.3** | **14373.2** | **18.1** | **46.0** | **33.3** | **6.3** | **14312.2** | **22.3** | **49.7** | **26.2** | **4.4** | **6945.3** | **36.0** | **40.6** | **21.3** | **5.0** | **672.8** | **48.9** | **30.1** | **17.5** | **8.2** |

Table A.3. Distribution of educational attainment by country, source, and age for men

| **Country** | **Source** | **Total Person-Years** | **Age 25-44** | | | | | **Age 45-64** | | | | | **Age 65-84** | | | | | **Age 85+** | | | | |
| --- | --- | --- | --- | --- | --- | --- | --- | --- | --- | --- | --- | --- | --- | --- | --- | --- | --- | --- | --- | --- | --- | --- |
|  |  | **(1,000,000s)** | **Person-Years** | **Low** | **Middle** | **High** | **Missing** | **Person-Years** | **Low** | **Middle** | **High** | **Missing** | **Person-Years** | **Low** | **Middle** | **High** | **Missing** | **Person-Years** | **Low** | **Middle** | **High** | **Missing** |
|  |  |  | **(1000s)** | **(%)** | **(%)** | **(%)** | **(%)** | **(1000s)** | **(%)** | **(%)** | **(%)** | **(%)** | **(1000s)** | **(%)** | **(%)** | **(%)** | **(%)** | **(1000s)** | **(%)** | **(%)** | **(%)** | **(%)** |
| AUS | OECD | 8.4 | 3,438 | 9.9 | 42.7 | 38.8 | 8.5 | 3,028 | 27.6 | 39.4 | 23.2 | 9.9 | 1,653 | 46.3 | 24.8 | 11.5 | 17.3 | 303 | 50.5 | 15.8 | 4.2 | 30 |
| AUT | OECD | 3.4 | 1,163 | 18 | 45 | 37 |  | 1,269 | 27.1 | 52 | 21 |  | 773 | 47.8 | 43 | 9.2 |  | 154 | 63.2 | 30.4 | 6.4 |  |
| CAN | OECD | 46.3 | 14,435 | 7.7 | 29.2 | 63.2 |  | 19,942 | 12.8 | 39.1 | 48.1 |  | 10,299 | 30.8 | 37.3 | 31.8 |  | 1,612 | 48.7 | 32.9 | 18.4 |  |
| DNK | OECD | 10.1 | 3,519 | 14 | 35.8 | 47.6 | 2.6 | 3,750 | 22.9 | 41.3 | 34.4 | 1.4 | 2,464 | 43.3 | 35.2 | 19.7 | 1.8 | 394 | 57.3 | 19.3 | 9 | 14 |
| ESP | OECD | 54.4 | 19,414 | 30.9 | 21.1 | 48 |  | 19,890 | 47.9 | 21.6 | 30.5 |  | 12,262 | 81 | 9.6 | 9.4 |  | 2,857 | 90.8 | 5.1 | 4.1 |  |
| EST | Eurostat | 2.1 | 724 | 10.3 | 39.5 | 49.3 | 0.8 | 733 | 6.7 | 45.1 | 47.7 | 0.5 | 569 | 27.5 | 40.3 | 31.6 | 0.7 | 98 | 54.7 | 26.3 | 17.9 | 1 |
| FIN | Eurostat | 12.2 | 3,975 | 11.4 | 39.4 | 49.2 |  | 4,436 | 15.7 | 41.5 | 42.7 |  | 3,185 | 48.2 | 29.9 | 21.9 |  | 573 | 74.8 | 15.5 | 9.7 |  |
| HUN | OECD | 15.2 | 5,069 | 11.6 | 50.7 | 37.7 |  | 5,491 | 20.7 | 56.4 | 22.9 |  | 4,144 | 48.5 | 37.9 | 13.7 |  | 529 | 80.1 | 13.1 | 6.9 |  |
| ITA | OECD | 91.1 | 26,719 | 29.3 | 44.7 | 26 |  | 34,418 | 50.2 | 36.2 | 13.6 |  | 24,606 | 81.6 | 13.5 | 4.9 |  | 5,335 | 89.5 | 8 | 2.5 |  |
| JPN | OECD |  |  |  |  |  |  |  |  |  |  |  |  |  |  |  |  |  |  |  |  |  |
| KOR | OECD | 71.7 | 25,894 | 1.1 | 26.9 | 72 |  | 31,036 | 24.7 | 47.5 | 27.8 |  | 14,264 | 78.4 | 15.8 | 5.8 |  | 472 | 92.1 | 6.1 | 1.8 |  |
| LTU | OECD | 5.8 | 1,794 | 13.8 | 41.5 | 44.8 |  | 2,208 | 4 | 56.3 | 39.7 |  | 1,572 | 32.5 | 48.9 | 18.5 |  | 266 | 68.1 | 21.4 | 10.5 |  |
| NLD | OECD | 0 | 0 |  |  |  |  | 0 |  |  |  |  | 0 |  |  |  |  | 0 |  |  |  |  |
| NOR | Eurostat | 10.8 | 4,129 | 15.5 | 27.2 | 50.9 | 6.4 | 3,879 | 21.6 | 41.1 | 35.7 | 1.7 | 2,297 | 31.9 | 48.5 | 18.9 | 0.7 | 459 | 49.3 | 40.7 | 9.2 | 1 |
| NZL | OECD | 22.8 | 8,708 | 18.2 | 30 | 40.6 | 11.2 | 8,854 | 31.6 | 27 | 30 | 11.4 | 4,549 | 45.4 | 17.8 | 18.1 | 18.8 | 703 | 44.8 | 17.1 | 10.3 | 28 |
| POL | OECD | 84 | 30,704 | 5.2 | 49.2 | 36.8 | 8.8 | 32,534 | 14.7 | 65.5 | 16 | 3.9 | 18,224 | 48.3 | 40.8 | 9 | 1.9 | 2,586 | 75.7 | 16.5 | 3.5 | 4 |
| SVK | OECD | 8.3 | 3,335 | 5.7 | 55.1 | 39.2 |  | 2,969 | 11.8 | 69.5 | 18.8 |  | 1,795 | 31.8 | 58.2 | 10 |  | 224 | 57.4 | 37.9 | 4.7 |  |
| SVN | Eurostat | 3.2 | 1,101 | 7.5 | 45.5 | 47 |  | 1,180 | 25.8 | 50.2 | 24 |  | 761 | 52 | 37.4 | 10.6 |  | 138 | 64.4 | 30.4 | 5.1 |  |
| SWE | OECD | 14.3 | 5,108 | 8.6 | 40.6 | 48.4 | 2.4 | 4,890 | 11.8 | 50.6 | 36.8 | 0.8 | 3,580 | 29.9 | 43.3 | 25.4 | 1.4 | 676 | 56 | 30.1 | 11.5 | 2 |
| TUR | OECD | 46.6 | 23,795 | 56.2 | 21.4 | 21.4 | 1.1 | 15,702 | 79.8 | 11.4 | 7.4 | 1.4 | 6,438 | 91.4 | 3.8 | 2.6 | 2.2 | 679 | 91.9 | 3 | 1.3 | 4 |
| USA | OECD | 230.6 | 86,342 | 8.6 | 40.8 | 50.6 |  | 85,953 | 10.6 | 46.8 | 42.6 |  | 50,109 | 14.1 | 53.1 | 32.9 |  | 8,156 | 23.6 | 56.5 | 19.9 |  |
| Average | | 37.1 | 13468.3 | 14.9 | 38.2 | 44.7 | 5.2 | 14108.1 | 24.6 | 44.1 | 29.6 | 3.9 | 8177.2 | 47.9 | 33.6 | 16.1 | 5.6 | 1310.7 | 64.9 | 22.4 | 8.3 | 10.5 |

**Table A4. Used correction factors (ratios of CPS deaths to death certificates deaths)**

*Source:* classification ratios are obtained from Rostron and colleagues Table 4.^14^ OECD classification of US educational attainment is based on ISCED 2011.

### A.3. Calculated statistics

*Life expectancy*

Abridged life tables are used to calculate period life expectancy using the Chiang method.^16^ Pooling observations in 5-year age groups is advantageous as it leads to larger sample sizes, thereby decreasing volatility of mortality rates. Volatile mortality rates are likely to arise in higher age groups with lower exposure levels as small changes in the number of deaths results in large changes in mortality rates.

#### Age-standardised mortality rates

This paper relies on age-standardised mortality rates (ASMR) to account for individual country-level variations in population structures over time and to control for age as a confounder of the education-longevity relationship. Mortality rates are adjusted directly using the 2010 OECD standard population.

#### Age-at-death

We derive the lifespan distribution, *f*, from age 25 using the corrected mortality rates, *m* and the corresponding survival function, *S*, based on the probability of death for each age-sex-education group.

$$f_{i}\left( t \right)=m_{i}\left( t \right).S_{i}\left( t \right)$$

$$S_{i}\left( t \right)= \prod_{a=1}^{t-1} \left( 1-m_{i}\left( t \right) \right),t>25$$

$$S\left( 25 \right)=1$$

where *m_i_* is the mortality rate for subgroup *i* at age *t*. We interpolate 5-year abridged corrected mortality rates to reflect 1-year mortality rates and smooth the lifespan distribution. Lifespan distribution figures are available on request.

#### Absolute and relative gaps

Gaps are simple pairwise comparisons of longevity between two groups. In life expectancy estimates, the absolute (relative) gap refers to the difference (ratio) in life expectancy between the high and low education groups:

$$Absolute Gap=LifeExpectancy_{High}-LifeExpectancy_{Low}$$

$$Relative Gap=\frac{LifeExpectancy_{High}}{LifeExpectancy_{Low}}$$

In principle, other pairwise comparisons between education groups could also be conducted. We only present comparisons of low and high education groups for the sake of brevity.

When applied to standardised mortality rates, the same concepts are referred to as rate difference (RD) and rate ratio (RR), for absolute and relative inequality measurement, respectively.

$$Rate Difference={MortalityRate}_{High}-{MortalityRate}_{Low}$$

$$Rate Ratio=\frac{MortalityRate_{High}}{MortalityRate_{Low}}$$

The absolute value of the RD and the inverse of the RR are presented below to maintain consistency in results interpretation.

#### Slope and relative indices of Inequality

The slope and relative indices of Inequality (SII; RII) are used to account for the entire education distribution and to provide an overall assessment of inequality across education groups. For the SII, this is accomplished by regressing the longevity outcome on a fraction-ranked education weighted by the education distribution, using an ordinary least squares regression to measure absolute inequality and a logistic regression to measure relative inequality.^17^ While Poisson distributions may, strictly speaking, be better to model mortality rates according to some authors, we have used the normal distribution as in the previous analysis, and checked that the differences in results between the two methods are minimal.^11,12^ Thus, the SII is:

$$y=g_{\alpha}\left( x \right)=y_{0}+\alpha x$$

$$SII=\alpha=g_{\alpha}\left( 1 \right)-g_{\alpha}(0)$$

where α is the magnitude and sign of the linear association between *x* and *y*, the education and longevity variables, respectively, when education is fraction-ranked between 0 and 1. SII values greater than zero indicate that the more educated groups have greater longevity than less educated groups. Conversely, if the SII is less than zero, the less educated have greater longevity. A SII value of zero indicates no inequalities in longevity between education groups. For standardised mortality rates, the absolute value of the SII is presented to maintain consistency in results interpretation.

The RII is calculated as:

$$y=f_{\beta}\left( x \right)=y_{0}exp(\beta x)$$

$$RII=\exp\left( \beta\right)=\frac{f_{\beta}(1)}{f_{\beta}(0)}$$

where *exp(β)* is the magnitude of the linear association between *x* and *y*, the education and longevity variables, respectively, when education is fraction-ranked between 0 and 1. An RII estimate equal to one indicates no relative advantage between predicted low and high education groups. If the RII is greater than one, higher education is associated with relatively greater longevity and when RII is less than one, higher education is associated with relatively lesser longevity. For example, a RII of 1.1 indicates that the highest education group has 10%, or 1.1 times, greater longevity than the predicted lowest education group. For standardised mortality rates, the inverse of the RII is presented to maintain consistency in results interpretation.

#### Theil Index

The Theil Index is included to decompose overall inequality into within- and between-group inequalities in age-at-death, providing insights into the contribution of education to total inequalities in life expectancy. We calculate the Theil Index, *T*, according to van Raalte and colleagues:^6^

$$T=\frac{1}{l_{a}}\sum_{x=a}^{\omega} d_{x}\left[ \left( \frac{\overline{x_{x}}}{e_{a}} \right)\ln\left( \frac{\overline{x_{x}}}{e_{a}} \right) \right]$$

where *a* and *ω* are the youngest and oldest life table ages (25 and 120 in this case), *l_a_* is the initial population size, e*_a_* is the population average age at death, *d_x_* is the life table number of deaths, and $\bar{x}_{x}$ is the average age at death in the age interval *x* to *x+n*.

The Theil Index is a generalised entropy index, which varies from zero to infinity, where zero represents a state of perfect equality where every person dies at the same age. The further the Theil Index is from zero, the greater the total inequality in age-at-death. Between-group variation refers to differences in total variation in age-at-death associated with education groups. Within-group variation refers to all other variation associated with the many other predictors of age-at-death. Between- and within- group components, *BG* and *WG*, are then calculated as follows:

$${BG}^{T}=\sum_{i=1}^{n} \left[ w^{i}\left( \frac{e_{a}^{i}}{e_{a}^{t}} \right)\ln\left( \frac{e_{a}^{i}}{e_{a}^{t}} \right) \right]$$

$${WG}^{T}=\sum_{i=1}^{n} \left[ w^{i}T^{i}\left( \frac{e_{a}^{i}}{e_{a}^{t}} \right) \right]$$

where *w^i^* is the population share of education subgroup, *i*, $e_{a}^{i}$is the average age at death conditional upon survival to age 25 for subgroup *i*, and $e_{a}^{t}$ is this average age at death for all education groups combined.

ANNEX B. ADDITIONAL RESULTS

### B.1. Life expectancy and life expectancy gaps

Figure B.1-B.2 and Table B.1 show life expectancy at age 25 and 65, respectively, by country, sex, and education. Across the 21 primary analysis country-sources, the average life expectancy at age 25 among individuals with low education is 56.1 and 49.3 years for women and men, respectively. For middle education groups, the average life expectancy at 25 is 59.3 and 53.6 years for women and men, respectively. For high education groups the average life expectancy at 25 is 61.3 and 57.5 years for women and men, respectively. Countries with life expectancies in all education groups greater than corresponding OECD average for men and women are Australia, Italy, Norway, and Spain. Countries with life expectancies below OECD average for men and women in all education groups are Hungary, Poland, and USA.

Trends in life expectancy (gaps) at age 25 are mirrored by trends observed at age 65. Women in low, middle, and high education groups have life expectancies of 20.4, 21.7, and 23.0 years at age 65, respectively. Men in low, middle, and high education groups have life expectancies of 16.2, 17.8, and 20.0 years at age 65, respectively. Countries with life expectancy greater than OECD average for men and women in all education groups are the same as at age 25, with the addition of Korea. Countries with life expectancy below OECD average for men and women in all education groups are similar to life expectancy at age 25, although including Türkiye and excluding the United States.

Figure B.1. Life expectancy at age 25 by country, sex, and education around 2016


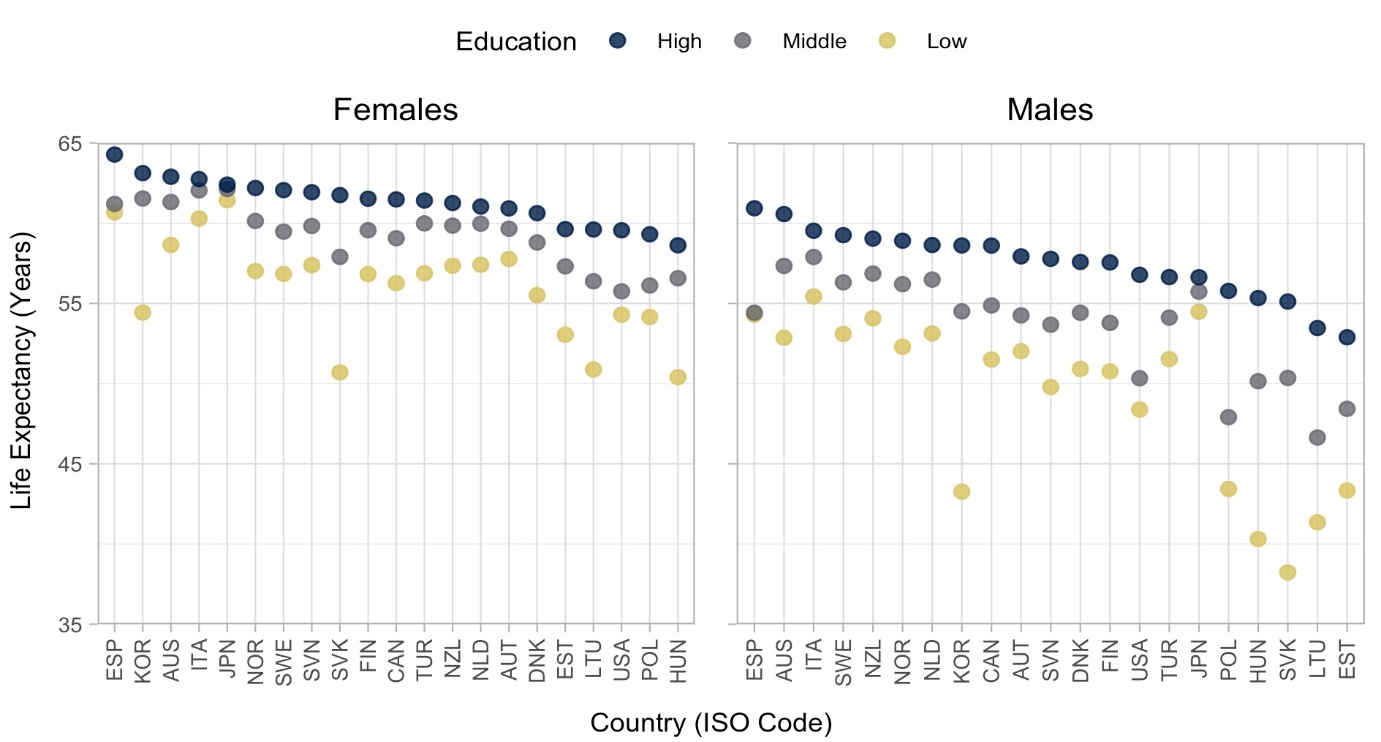


Note: Countries are reported in International Organization for Standardization (ISO) three-letter codes. Education is classified according to the 2011 International Standard Classification of Education (ISCED-2011) into low (lower secondary education and below, ISCED 0-2), medium (upper-secondary, ISCED 3-4), and high education (higher than upper-secondary, ISCED 5-8).

Figure B.2. Life expectancy at age 65 by country, sex, and education around 2016


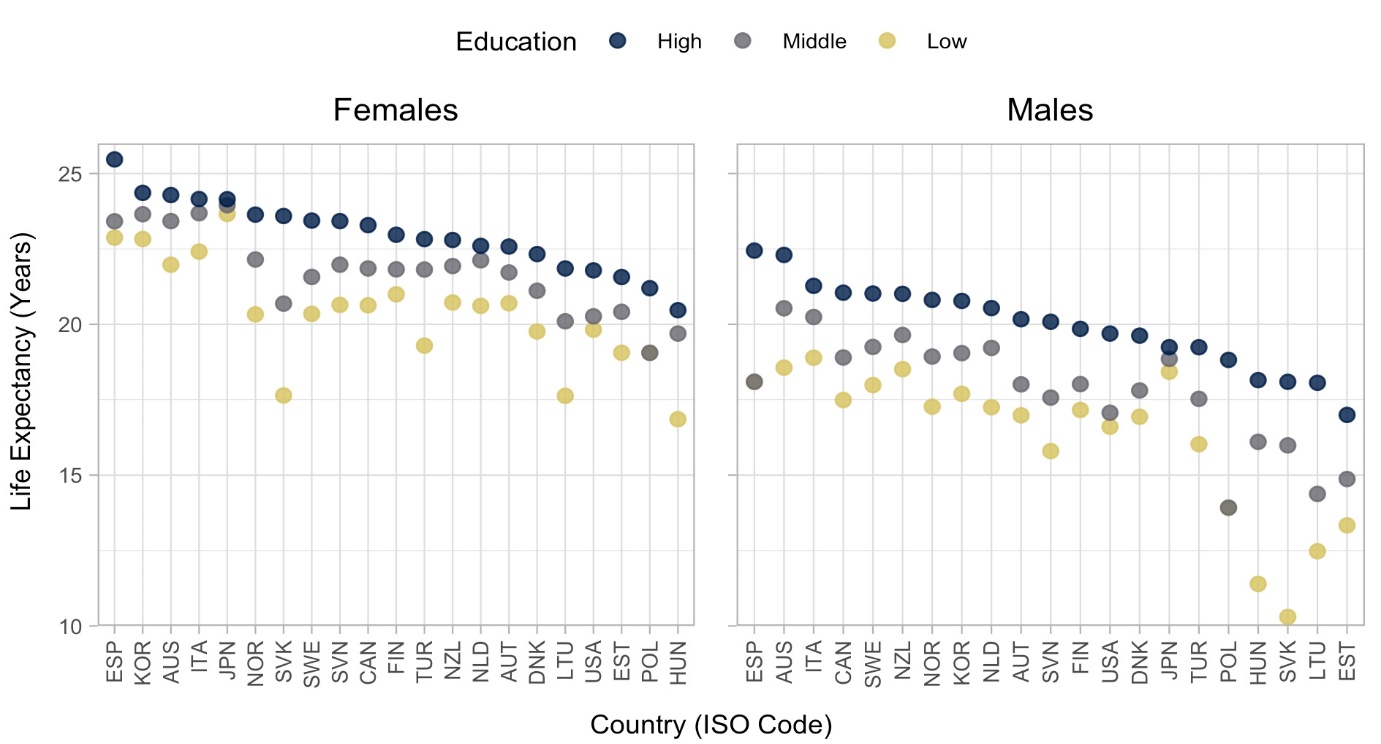


Note: Countries are reported in International Organization for Standardization (ISO) three-letter codes. Education is classified according to the 2011 International Standard Classification of Education (ISCED-2011) into low (lower secondary education and below, ISCED 0-2), medium (upper-secondary, ISCED 3-4), and high education (higher than upper-secondary, ISCED 5-8).

Table B.1. Life expectancy at 25 and 65 by country, sex, and education

| **Country** | **Source** | **Life expectancy at 25** | | | | | | **Life expectancy at 65** | | | | | |
| --- | --- | --- | --- | --- | --- | --- | --- | --- | --- | --- | --- | --- | --- |
|  |  | **Females** | | | **Males** | | | **Females** | | | **Males** | | |
|  |  | **Low** | **Middle** | **High** | **Low** | **Middle** | **High** | **Low** | **Middle** | **High** | **Low** | **Middle** | **High** |
| AUS | OECD | 58.6 | 61.3 | 62.9 | 52.9 | 57.3 | 60.6 | 22 | 23.4 | 24.3 | 18.6 | 20.5 | 22.3 |
| AUT | OECD | 57.8 | 59.7 | 60.9 | 52 | 54.3 | 57.9 | 20.7 | 21.7 | 22.6 | 17 | 18 | 20.2 |
| CAN | OECD | 56.3 | 59.1 | 61.5 | 51.5 | 54.9 | 58.6 | 20.6 | 21.9 | 23.3 | 17.5 | 18.9 | 21 |
| DNK | OECD | 55.5 | 58.8 | 60.6 | 50.9 | 54.4 | 57.6 | 19.8 | 21.1 | 22.3 | 16.9 | 17.8 | 19.6 |
| ESP | OECD | 60.7 | 61.2 | 64.2 | 54.3 | 54.4 | 60.9 | 22.9 | 23.4 | 25.5 | 18.1 | 18.1 | 22.4 |
| EST | Eurostat | 53 | 57.3 | 59.6 | 43.3 | 48.4 | 52.9 | 19.1 | 20.4 | 21.6 | 13.3 | 14.9 | 17 |
| FIN | Eurostat | 56.8 | 59.6 | 61.5 | 50.8 | 53.8 | 57.6 | 21 | 21.8 | 23 | 17.2 | 18 | 19.8 |
| HUN | OECD | 50.4 | 56.6 | 58.6 | 40.3 | 50.1 | 55.3 | 16.9 | 19.7 | 20.5 | 11.4 | 16.1 | 18.1 |
| ITA | OECD | 60.3 | 62 | 62.7 | 55.4 | 57.9 | 59.5 | 22.4 | 23.7 | 24.2 | 18.9 | 20.2 | 21.3 |
| JPN | OECD | 61.4 | 62.1 | 62.4 | 54.5 | 55.7 | 56.6 | 23.7 | 23.9 | 24.1 | 18.4 | 18.9 | 19.2 |
| KOR | OECD | 54.4 | 61.5 | 63.1 | 43.3 | 54.5 | 58.6 | 22.8 | 23.7 | 24.4 | 17.7 | 19 | 20.8 |
| LTU | OECD | 50.9 | 56.4 | 59.6 | 41.4 | 46.6 | 53.5 | 17.6 | 20.1 | 21.9 | 12.5 | 14.4 | 18.1 |
| NLD | OECD | 57.4 | 60 | 61 | 53.1 | 56.5 | 58.6 | 20.6 | 22.1 | 22.6 | 17.3 | 19.2 | 20.5 |
| NOR | Eurostat | 57 | 60.1 | 62.2 | 52.3 | 56.2 | 58.9 | 20.3 | 22.1 | 23.6 | 17.3 | 18.9 | 20.8 |
| NZL | OECD | 57.3 | 59.8 | 61.3 | 54.1 | 56.9 | 59 | 20.7 | 21.9 | 22.8 | 18.5 | 19.6 | 21 |
| POL | OECD | 54.2 | 56.1 | 59.3 | 43.4 | 47.9 | 55.8 | 19.1 | 19.1 | 21.2 | 13.9 | 13.9 | 18.8 |
| SVK | OECD | 50.7 | 57.9 | 61.7 | 38.2 | 50.4 | 55.1 | 17.6 | 20.7 | 23.8 | 10.3 | 16 | 18.1 |
| SVK | Eurostat | 51 | 57.3 | 60.9 | 38.6 | 50.1 | 54.8 | 17.7 | 20 | 22.7 | 10.4 | 15.6 | 17.8 |
| SVN | Eurostat | 57.4 | 59.8 | 61.9 | 49.8 | 53.7 | 57.8 | 20.6 | 22 | 23.4 | 15.8 | 17.6 | 20.1 |
| SWE | OECD | 56.8 | 59.5 | 62.1 | 53.1 | 56.3 | 59.3 | 20.3 | 21.6 | 23.4 | 18 | 19.2 | 21 |
| TUR | OECD | 56.9 | 60 | 61.4 | 51.5 | 54.1 | 56.6 | 19.2 | 21.8 | 22.8 | 16 | 17.5 | 19.2 |
| USA | OECD | 54.3 | 55.8 | 59.6 | 48.4 | 50.3 | 56.8 | 19.8 | 20.3 | 21.8 | 16.6 | 17.1 | 19.7 |
| **Average** | | **55.9** | **59.2** | **61.3** | **48.8** | **53.4** | **57.4** | **20.2** | **21.7** | **23.0** | **16.0** | **17.7** | **19.9** |

Absolute gaps in life expectancy at age 65 for men and women are 4.1 and 3.1 years, respectively, a decrease of 4.5 years (55%) for men and 2.6 years (50%) for women relative to those observed at age 25 (Figure B.3 and Table B.2). Relative gaps in life expectancy at age 65 for women and men are 1.113 and 1.251, respectively, an increase of 0.039 for women and 0.073 for men from relative life expectancy gaps at age 25. Country-source ranks for absolute and relative gaps are mostly similar at age 25 and 65. The largest outlier is the change in gap ranks for Korea, where the relative gap is third highest for men and women at age 25, but is second lowest for women and eighth lowest for men at age 65.

Figure B.3. Absolute and relative life expectancy gaps between high and low educated people at age 65 by country and sex around 2016


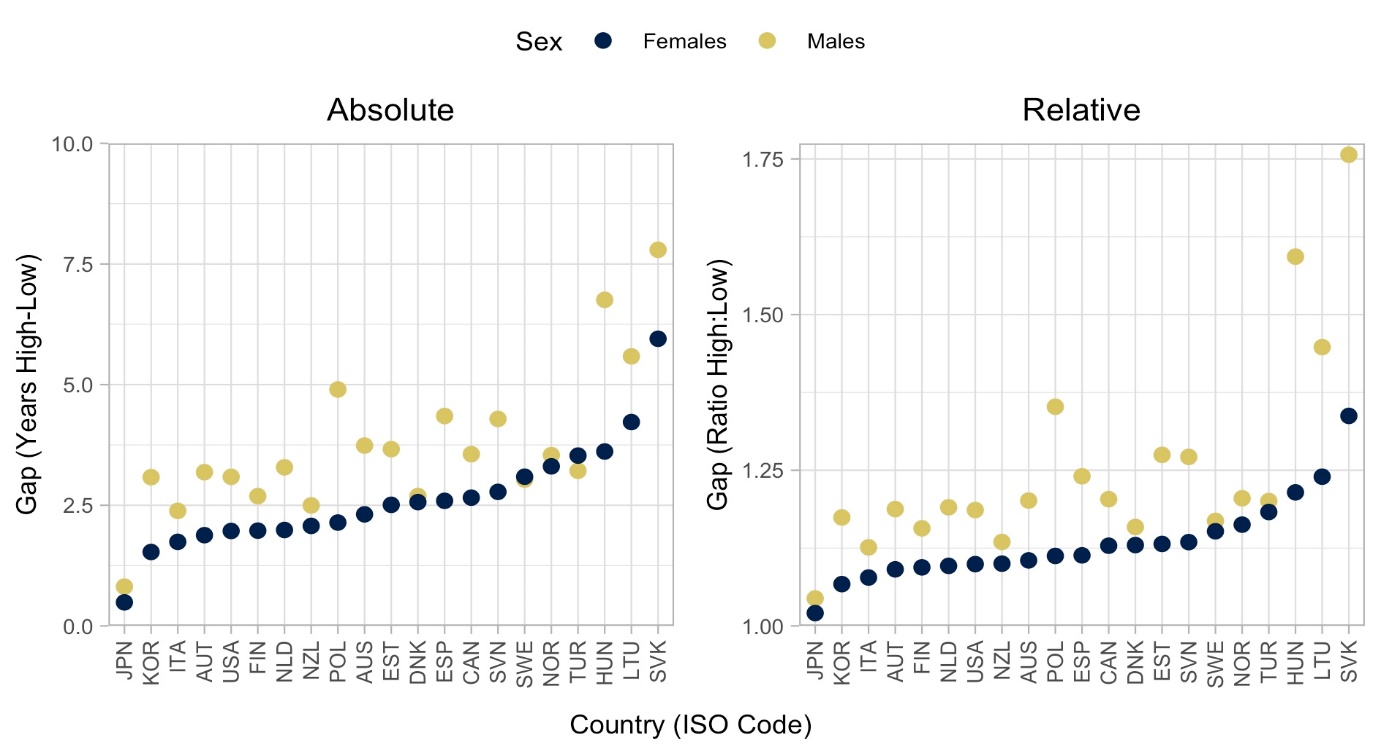


Note: Countries are reported in International Organization for Standardization (ISO) three-letter codes.

Table B.2. Absolute and relative gaps in life expectancy at 25 and 65 by country, sex, and education

| **Country** | **Source** | **Life Expectancy at 25** | | | | **Life Expectancy at 65** | | | |
| --- | --- | --- | --- | --- | --- | --- | --- | --- | --- |
|  |  | **Absolute Gap** | | **Relative Gap** | | **Absolute Gap** | | **Relative Gap** | |
|  |  | **Females** | **Males** | **Females** | **Males** | **Females** | **Males** | **Females** | **Males** |
| AUS | OECD | 4.2 | 7.7 | 1.072 | 1.146 | 2.3 | 3.7 | 1.105 | 1.201 |
| AUT | OECD | 3.2 | 5.9 | 1.055 | 1.114 | 1.9 | 3.2 | 1.091 | 1.188 |
| CAN | OECD | 5.2 | 7.1 | 1.093 | 1.138 | 2.7 | 3.6 | 1.129 | 1.204 |
| DNK | OECD | 5.1 | 6.7 | 1.092 | 1.131 | 2.6 | 2.7 | 1.13 | 1.159 |
| ESP | OECD | 3.6 | 6.6 | 1.059 | 1.122 | 2.6 | 4.3 | 1.113 | 1.24 |
| EST | Eurostat | 6.6 | 9.5 | 1.124 | 1.22 | 2.5 | 3.7 | 1.132 | 1.275 |
| FIN | Eurostat | 4.7 | 6.8 | 1.083 | 1.134 | 2 | 2.7 | 1.094 | 1.157 |
| HUN | OECD | 8.2 | 15 | 1.163 | 1.373 | 3.6 | 6.8 | 1.214 | 1.593 |
| ITA | OECD | 2.5 | 4.1 | 1.041 | 1.074 | 1.7 | 2.4 | 1.078 | 1.126 |
| JPN | OECD | 1 | 2.2 | 1.016 | 1.039 | 0.5 | 0.8 | 1.021 | 1.044 |
| KOR | OECD | 8.7 | 15.3 | 1.16 | 1.355 | 1.5 | 3.1 | 1.067 | 1.174 |
| LTU | OECD | 8.7 | 12.1 | 1.172 | 1.293 | 4.2 | 5.6 | 1.24 | 1.448 |
| NLD | OECD | 3.6 | 5.5 | 1.063 | 1.104 | 2 | 3.3 | 1.096 | 1.191 |
| NOR | Eurostat | 5.2 | 6.6 | 1.091 | 1.126 | 3.3 | 3.5 | 1.163 | 1.205 |
| NZL | OECD | 3.9 | 5 | 1.068 | 1.092 | 2.1 | 2.5 | 1.1 | 1.135 |
| POL | OECD | 5.2 | 12.3 | 1.095 | 1.284 | 2.1 | 4.9 | 1.112 | 1.352 |
| SVK | OECD | 11.1 | 16.9 | 1.218 | 1.442 | 6 | 7.8 | 1.337 | 1.757 |
| SVN | Eurostat | 4.6 | 8 | 1.079 | 1.161 | 2.8 | 4.3 | 1.135 | 1.271 |
| SWE | OECD | 5.2 | 6.2 | 1.092 | 1.116 | 3.1 | 3 | 1.152 | 1.169 |
| TUR | OECD | 4.5 | 5.1 | 1.08 | 1.099 | 3.5 | 3.2 | 1.183 | 1.201 |
| USA | OECD | 5.3 | 8.4 | 1.097 | 1.174 | 2 | 3.1 | 1.099 | 1.186 |
| **Average** | | **5.3** | **8.2** | **1.1** | **1.2** | **2.6** | **3.7** | **1.1** | **1.3** |

### B.2. Age-standardised mortality rates

Table B.3. reports age-standardised mortality rates per 100,000 adults older than 25 years, by country, sex and education.

Table B.3. Age-standardised mortality rates by country, sex, and education around 2016

| **Country** | **Source** | **Females** | | | **Males** | | |
| --- | --- | --- | --- | --- | --- | --- | --- |
|  |  | **Low** | **Middle** | **High** | **Low** | **Middle** | **High** |
| AUS | OECD | 1361 | 1213 | 1133 | 1766 | 1440 | 1239 |
| AUT | OECD | 1481 | 1370 | 1289 | 1961 | 1773 | 1494 |
| CAN | OECD | 1545 | 1366 | 1231 | 1930 | 1668 | 1407 |
| DNK | OECD | 1587 | 1367 | 1254 | 2026 | 1744 | 1496 |
| ESP | OECD | 1210 | 1189 | 1024 | 1678 | 1671 | 1191 |
| EST | Eurostat | 1792 | 1498 | 1347 | 2897 | 2336 | 1890 |
| FIN | Eurostat | 1520 | 1342 | 1232 | 2015 | 1780 | 1496 |
| HUN | OECD | 2138 | 1634 | 1461 | 3571 | 2196 | 1712 |
| ITA | OECD | 1177 | 1055 | 1022 | 1514 | 1332 | 1225 |
| KOR | OECD | 1574 | 1217 | 1133 | 2511 | 1696 | 1411 |
| LTU | OECD | 1978 | 1549 | 1331 | 3198 | 2481 | 1785 |
| NLD | OECD | 1509 | 1348 | 1290 | 1881 | 1622 | 1427 |
| NOR | Eurostat | 1476 | 1281 | 1171 | 1894 | 1590 | 1401 |
| NZL | OECD | 1464 | 1310 | 1230 | 1731 | 1527 | 1382 |
| POL | OECD | 1625 | 1517 | 1292 | 2662 | 2314 | 1536 |
| SVK | OECD | 1917 | 1359 | 1062 | 3985 | 2170 | 1736 |
| SVN | Eurostat | 1465 | 1304 | 1183 | 2144 | 1781 | 1446 |
| SWE | OECD | 1483 | 1314 | 1171 | 1785 | 1542 | 1342 |
| TUR | OECD | 1477 | 1277 | 1131 | 1949 | 1708 | 1494 |
| USA | OECD | 1682 | 1579 | 1335 | 2236 | 2064 | 1558 |
| **Average** | | **1573** | **1354** | **1216** | **2267** | **1822** | **1483** |

### B.3. Slope Index of Inequality and Relative Index of Inequality

Figure B.4 shows the SII and RII results for standardised mortality rates; the sample average SII for women and men is approximately 450 and 950 deaths per 100 000, respectively. The sample average RII for women and men is approximately 1.40 for women and 1.69 for men. The SII and RII estimates indicate 26% and 12% greater inequality than those obtained using the RD and RR, respectively. Australia, Austria, Italy, and Spain consistently rank lowest in absolute and relative measures of inequality, whereas Estonia, Hungary, Lithuania, and the Slovak Republic consistently rank among the highest inequality countries.

**Figure B.4. Slope and relative indices of inequality in age-standardised mortality rates by country and sex around 2016**


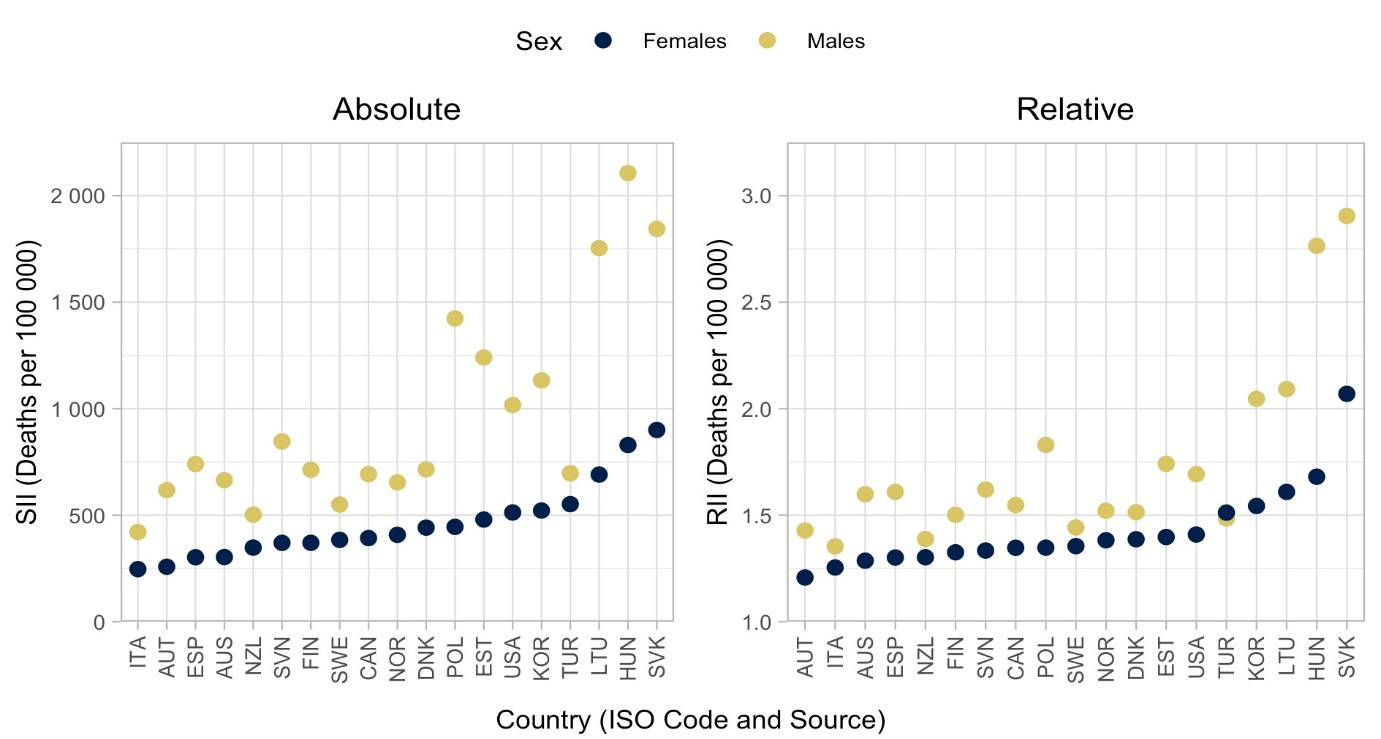


Note: SII, slope index of inequality; RII, relative index of inequality. Countries are reported in International Organization for Standardization (ISO) three-letter codes. Mortality rates are standardised using the OECD 2010 standard population.

### B.4. Standard deviations of lifespan distributions

Figure B.5 shows the standard deviations (SDs) of lifespan distributions in age-at-death by country, sex, and education (full results available in Table B.4). The sample average SD for women in low, middle, and high education groups is 14.0, 12.1, and 10.8 years, respectively, with a mean age-at-death of 82.0, 85.4, and 87.5 years, respectively. For men, the lifespan SD for low, middle, and high education groups is 14.9, 13.3, and 11.6 years, respectively, with a with a mean age-at-death of 75.0, 79.7, and 83.8 years, respectively.

In Canada, Lithuania, and the United States, the lifespan SD is greater than the sample average across all education groups for both men and women. Women and men in the United States with middle educational attainment have the highest lifespan SD of any country in the sample, at 14.4 and 15.6 years, respectively. The lifespan SD for Koreans with low educational attainment is especially large; 20 years for women and 22 years for men, which is 4.4 and 3.7 years greater than the second-highest SD for women (Slovak Republic) and men (Poland), respectively.

Figure B.5. Standard deviations in age-at-death by country, sex, and education around 2016


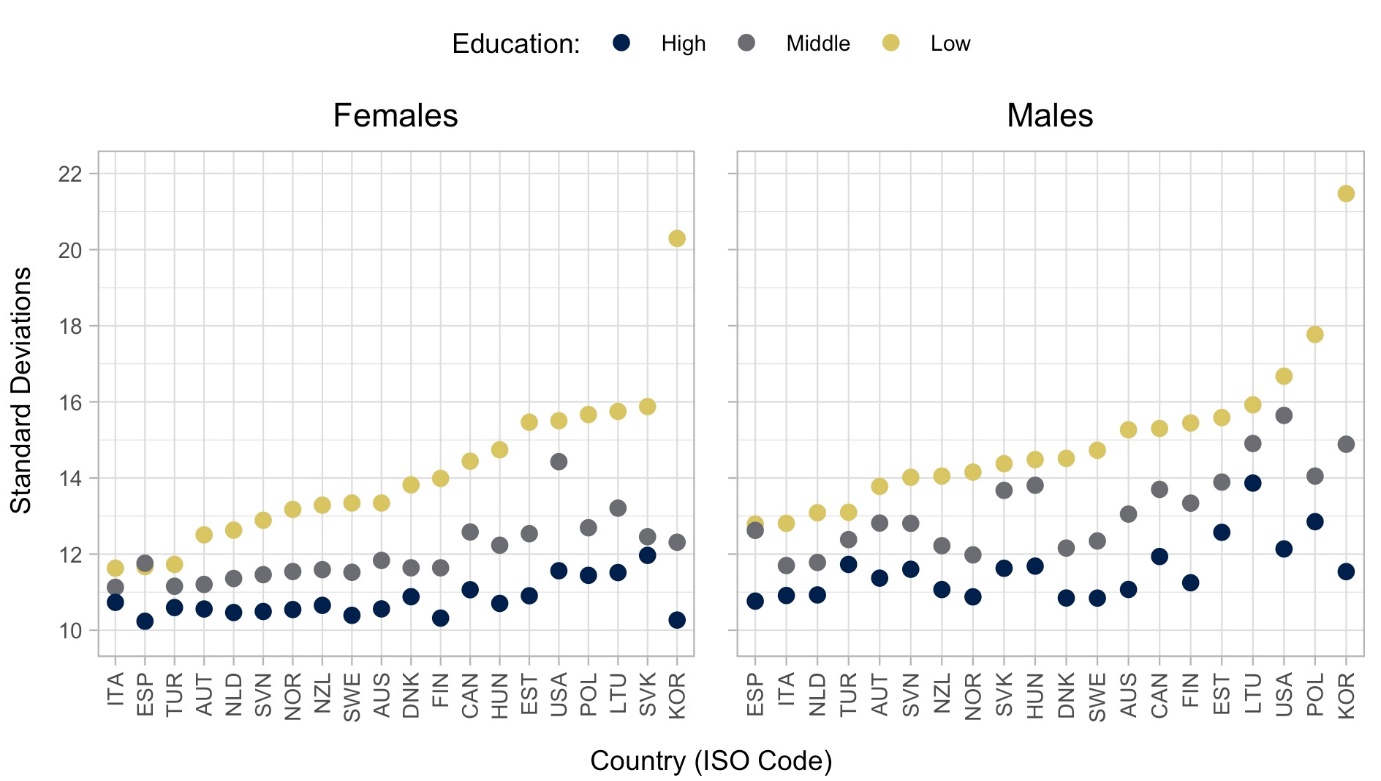


Note: Countries are reported in International Organization for Standardization (ISO) three-letter codes. Mortality rates are standardised using the OECD 2010 standard population.

Table B.4. Mean and standard deviation of age-at-death by country, sex, and education

| **Country** | **Source** | **Females** | | | | | | **Males** | | | | | |
| --- | --- | --- | --- | --- | --- | --- | --- | --- | --- | --- | --- | --- | --- |
|  |  | **Low** | | **Middle** | | **High** | | **Low** | | **Middle** | | **High** | |
|  |  | **Mean** | **SD** | **Mean** | **SD** | **Mean** | **SD** | **Mean** | **SD** | **Mean** | **SD** | **Mean** | **SD** |
| AUS | OECD | 84.7 | 13.3 | 87.3 | 11.8 | 88.9 | 10.6 | 78.8 | 15.3 | 83.3 | 13.1 | 86.6 | 11.1 |
| AUT | OECD | 83.7 | 12.5 | 85.7 | 11.2 | 86.9 | 10.6 | 78 | 13.8 | 80.3 | 12.8 | 83.9 | 11.4 |
| BEL | UN | 82.4 | 13.9 | 86.1 | 14.1 | 90.8 | 11.5 | 77.7 | 14.1 | 78.3 | 13.4 | 85.9 | 10.8 |
| CAN | OECD | 82.3 | 14.4 | 85.1 | 12.6 | 87.5 | 11.1 | 77.4 | 15.3 | 80.8 | 13.7 | 84.6 | 11.9 |
| CZE | UN | 77.8 | 13.3 | 82.5 | 11.3 | 83.7 | 11.8 | 72.4 | 14.9 | 77.5 | 12.8 | 78.6 | 12.3 |
| DNK | OECD | 81.6 | 13.8 | 84.8 | 11.6 | 86.7 | 10.9 | 76.9 | 14.5 | 80.5 | 12.2 | 83.6 | 10.8 |
| ESP | OECD | 86.7 | 11.7 | 87.2 | 11.8 | 90.3 | 10.2 | 80.3 | 12.8 | 80.5 | 12.6 | 87 | 10.8 |
| ESP | UN | 87.2 | 12.9 | 87.6 | 12.4 | 89.7 | 12.8 | 79.8 | 13 | 80.5 | 12 | 83.9 | 12 |
| EST | Eurostat | 79 | 15.5 | 83.4 | 12.5 | 85.7 | 10.9 | 69.4 | 15.6 | 74.5 | 13.9 | 79 | 12.6 |
| EST | UN | 76.2 | 15.1 | 84.3 | 12.9 | 86.1 | 11.2 | 70.1 | 16.1 | 74.5 | 13.6 | 77.6 | 12.4 |
| FIN | Eurostat | 82.8 | 14 | 85.6 | 11.6 | 87.5 | 10.3 | 76.6 | 15.4 | 79.8 | 13.3 | 83.6 | 11.2 |
| GRC | UN | 85.7 | 12.7 | 87.6 | 11.8 | 88.3 | 10.8 | 77.9 | 15.5 | 82.2 | 15 | 85.7 | 12.7 |
| HUN | OECD | 76.4 | 14.7 | 82.6 | 12.2 | 84.6 | 10.7 | 66.2 | 14.5 | 76.2 | 13.8 | 81.3 | 11.7 |
| HUN | UN | 77 | 14.2 | 82.4 | 12.2 | 83.8 | 11 | 66.7 | 13.2 | 76.6 | 14.3 | 80.4 | 12.6 |
| ITA | OECD | 86.3 | 11.6 | 88.1 | 11.1 | 88.8 | 10.7 | 81.4 | 12.8 | 83.9 | 11.7 | 85.6 | 10.9 |
| KOR | OECD | 80 | 20.3 | 87.4 | 12.3 | 89.1 | 10.3 | 68.3 | 21.5 | 80.2 | 14.9 | 84.6 | 11.5 |
| LTU | OECD | 76.8 | 15.7 | 82.4 | 13.2 | 85.6 | 11.5 | 67.2 | 15.9 | 72.7 | 14.9 | 79.5 | 13.9 |
| NLD | OECD | 83.4 | 12.6 | 86 | 11.4 | 87 | 10.5 | 79.1 | 13.1 | 82.5 | 11.8 | 84.6 | 10.9 |
| NOR | Eurostat | 83 | 13.2 | 86.1 | 11.5 | 88.2 | 10.5 | 78.3 | 14.2 | 82.2 | 12 | 84.9 | 10.9 |
| NOR | UN | 83.3 | 13.6 | 86.6 | 12.2 | 88.8 | 11.4 | 79.1 | 14.3 | 83.1 | 12.4 | 85.5 | 12 |
| NZL | OECD | 83.4 | 13.3 | 85.9 | 11.6 | 87.3 | 10.7 | 80 | 14.1 | 82.9 | 12.2 | 85.1 | 11.1 |
| POL | OECD | 79.9 | 15.7 | 82.1 | 12.7 | 85.3 | 11.4 | 68.8 | 17.8 | 73.8 | 14.1 | 81.8 | 12.9 |
| POL | UN | 80.2 | 14.9 | 83 | 12.6 | 87.3 | 11.8 | 69.9 | 16.8 | 75.1 | 14 | 82.5 | 13.2 |
| PRT | UN | 85.4 | 12.3 | 90.6 | 12.8 | 92.1 | 11.5 | 78.6 | 14.3 | 85.2 | 15 | 88.4 | 12.6 |
| SVK | OECD | 76.8 | 15.9 | 83.9 | 12.5 | 87.8 | 12 | 64.1 | 14.4 | 76.4 | 13.7 | 81.1 | 11.6 |
| SVK | Eurostat | 77.1 | 15.6 | 83.4 | 12.1 | 86.9 | 11.6 | 64.5 | 14.4 | 76.1 | 13.5 | 80.9 | 11.6 |
| SVN | Eurostat | 83.4 | 12.9 | 85.9 | 11.5 | 87.9 | 10.5 | 75.9 | 14 | 79.7 | 12.8 | 83.8 | 11.6 |
| SVN | UN | 82.7 | 12 | 84.9 | 10.7 | 87.1 | 9.8 | 75.8 | 13.5 | 79.4 | 12.2 | 83.2 | 10.8 |
| SWE | OECD | 82.8 | 13.3 | 85.5 | 11.5 | 88.1 | 10.4 | 78.9 | 14.7 | 82.3 | 12.3 | 85.3 | 10.8 |
| SWE | Eurostat | 83.5 | 12.9 | 85.5 | 11.4 | 87.8 | 10.2 | 79.3 | 14.3 | 82.2 | 12.2 | 85 | 10.6 |
| SWE | UN | 79.8 | 13.5 | 87.8 | 11.5 | 88.3 | 10.7 | 77.6 | 14.9 | 83 | 12.3 | 85.3 | 11.1 |
| TUR | OECD | 82.9 | 11.7 | 86 | 11.2 | 87.4 | 10.6 | 77.5 | 13.1 | 80.1 | 12.4 | 82.7 | 11.7 |
| USA | OECD | 80.2 | 15.5 | 81.8 | 14.4 | 85.6 | 11.6 | 74.2 | 16.7 | 76.3 | 15.6 | 82.8 | 12.1 |
| **Average** | | **81.6** | **13.9** | **85.3** | **12.1** | **87.5** | **11** | **74.7** | **14.8** | **79.5** | **13.2** | **83.5** | **11.7** |

### B.5. Theil index of lifespan distributions

Figure B.6 shows the total Theil index and the between- and within-group components (complete results available in Table B.5). The sample average Theil Index is 1.23 for women and 1.73 for men, while the within-components are 1.18 and 1.57 for women and men, respectively. Consequently, the between-group component accounts for 3.8 and 7.5% of the total Theil Index for women and men, respectively. This low share is consistent with the large gaps in life expectancy between education groups as the dispersion of ages at death within groups is still very large.

There is considerable variation in country ranks between the total Theil Index and the between-group relative component for country-sex groups. The United States has the fourth highest total Theil Index and the ninth lowest between-group component. Nevertheless, some country-specific trends emerge; Italy records the lowest total Theil Index and between-group component among all countries in the sample. The Slovak Republic has the highest between-group components, accounting for 13% and 30% of the Theil Index for women and men, respectively.

Figure B.6. Theil index of inequality in age-at-death by country and sex around 2016


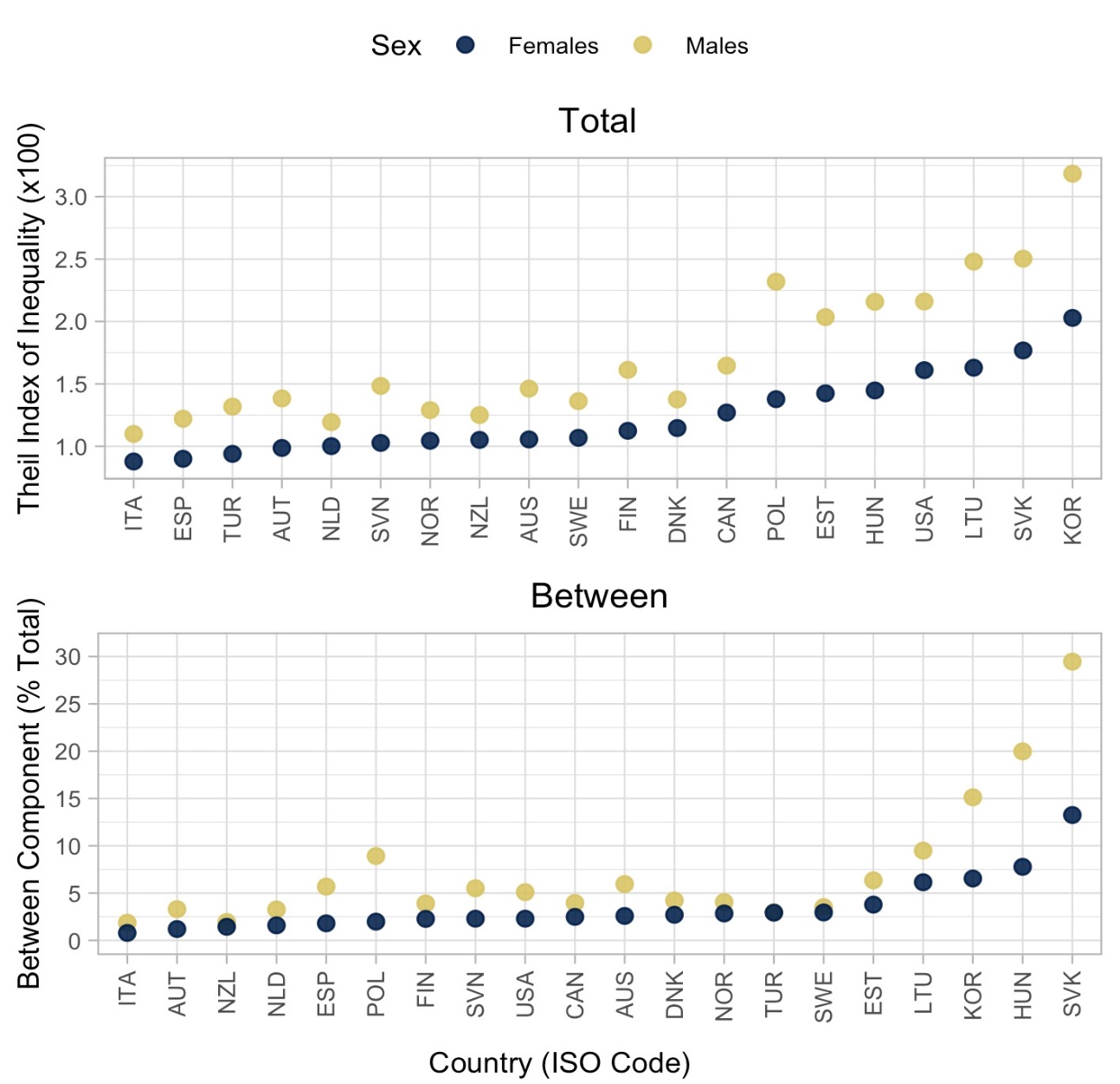


Note: Countries are reported in International Organization for Standardization (ISO) three-letter codes.

Table B.5. Theil index of inequality in age-at-death by country and sex

| **Country** | **Source** | **Females** | | | | **Males** | | | | **All** | | | |
| --- | --- | --- | --- | --- | --- | --- | --- | --- | --- | --- | --- | --- | --- |
|  |  | **Theil Index** | **Within** | **Between** | **Between** | **Theil Index** | **Within** | **Between** | **Between** | **Theil Index** | **Within** | **Between** | **Between** |
|  |  | **(x100)** |  |  | **(% Total)** | **(x100)** |  |  | **(% Total)** | **(x100)** |  |  | **(% Total)** |
| AUS | OECD | 1.055 | 1.028 | 0.027 | 2.6 | 1.462 | 1.375 | 0.087 | 6 | 1.281 | 1.229 | 0.052 | 4.1 |
| AUT | OECD | 0.987 | 0.975 | 0.012 | 1.2 | 1.384 | 1.338 | 0.046 | 3.3 | 1.22 | 1.195 | 0.025 | 2.1 |
| CAN | OECD | 1.27 | 1.239 | 0.032 | 2.5 | 1.646 | 1.581 | 0.065 | 4 | 1.483 | 1.436 | 0.046 | 3.1 |
| DNK | OECD | 1.146 | 1.115 | 0.031 | 2.7 | 1.376 | 1.318 | 0.058 | 4.2 | 1.288 | 1.245 | 0.043 | 3.3 |
| ESP | OECD | 0.9 | 0.883 | 0.016 | 1.8 | 1.221 | 1.151 | 0.069 | 5.7 | 1.107 | 1.069 | 0.037 | 3.4 |
| EST | Eurostat | 1.424 | 1.371 | 0.054 | 3.8 | 2.035 | 1.906 | 0.129 | 6.3 | 1.854 | 1.769 | 0.085 | 4.6 |
| FIN | Eurostat | 1.124 | 1.099 | 0.026 | 2.3 | 1.612 | 1.549 | 0.063 | 3.9 | 1.411 | 1.37 | 0.041 | 2.9 |
| HUN | OECD | 1.448 | 1.335 | 0.113 | 7.8 | 2.158 | 1.727 | 0.431 | 20 | 1.881 | 1.642 | 0.239 | 12.7 |
| ITA | OECD | 0.878 | 0.871 | 0.007 | 0.8 | 1.099 | 1.078 | 0.021 | 1.9 | 1.014 | 1.002 | 0.013 | 1.2 |
| KOR | OECD | 2.029 | 1.896 | 0.133 | 6.5 | 3.184 | 2.702 | 0.482 | 15.1 | 2.697 | 2.426 | 0.271 | 10 |
| LTU | OECD | 1.63 | 1.53 | 0.1 | 6.1 | 2.48 | 2.245 | 0.235 | 9.5 | 2.182 | 2.027 | 0.155 | 7.1 |
| NLD | OECD | 1.001 | 0.986 | 0.016 | 1.6 | 1.194 | 1.155 | 0.039 | 3.3 | 1.116 | 1.09 | 0.026 | 2.3 |
| NOR | Eurostat | 1.044 | 1.014 | 0.03 | 2.8 | 1.29 | 1.238 | 0.052 | 4 | 1.192 | 1.152 | 0.04 | 3.4 |
| NZL | OECD | 1.051 | 1.036 | 0.015 | 1.4 | 1.25 | 1.226 | 0.024 | 1.9 | 1.164 | 1.144 | 0.019 | 1.7 |
| POL | OECD | 1.378 | 1.35 | 0.027 | 2 | 2.319 | 2.112 | 0.207 | 8.9 | 1.939 | 1.847 | 0.092 | 4.7 |
| SVK | OECD | 1.768 | 1.534 | 0.234 | 13.3 | 2.503 | 1.765 | 0.738 | 29.5 | 2.29 | 1.855 | 0.435 | 19 |
| SVN | Eurostat | 1.027 | 1.004 | 0.024 | 2.3 | 1.484 | 1.402 | 0.082 | 5.5 | 1.312 | 1.264 | 0.047 | 3.6 |
| SWE | OECD | 1.068 | 1.037 | 0.032 | 3 | 1.362 | 1.314 | 0.048 | 3.5 | 1.231 | 1.192 | 0.039 | 3.2 |
| TUR | OECD | 0.939 | 0.912 | 0.028 | 2.9 | 1.318 | 1.279 | 0.039 | 3 | 1.174 | 1.142 | 0.033 | 2.8 |
| USA | OECD | 1.61 | 1.573 | 0.037 | 2.3 | 2.16 | 2.05 | 0.11 | 5.1 | 1.922 | 1.854 | 0.068 | 3.5 |
| **Average** | | **1.24** | **1.19** | **0.05** | **3.49** | **1.73** | **1.58** | **0.15** | **7.23** | **1.54** | **1.45** | **0.09** | **4.94** |

### B.6. Decompositions by causes of death

Table B.6 and Table B.7 show cause-specific standardised mortality rates by sex, education, and two age-groups (25-64 and 65-89). Among younger women, neoplasms have the highest standardised mortality rate for all education groups. Among older women, neoplasms have the highest standardised mortality rate in middle and high education groups, but not for low education groups, where standardised mortality rates due to circulatory system diseases are highest. For younger men, “Other” causes of death have the highest standardised mortality rate among low and middle education groups, whereas neoplasms have the highest standardised mortality rate for high education groups. For older men, circulatory deaths have the highest standardised mortality rate on average across the three education groups. External deaths have the lowest standardised mortality rate for women and men in both age groups. Among high all-cause standardised mortality rate countries, such as Hungary, Lithuania, Slovak Republic, and Poland, deaths from circulatory disease and other causes tend to be substantially greater than other countries.

Table B.8 and Table B.9 show the SII and RII of age-standardised mortality rates by country, age, and cause of death for women and men, respectively. SIIs tend to be greater among older adults and RIIs tend to be greater among younger adults. Among men and women in both age groups, RIIs tend to be consistently lowest in neoplasm deaths and consistently highest in circulatory deaths, with more variability in deaths by external and other causes. Among younger adults, SIIs and RIIs are highest or “Other” causes of death for both women and men and fall sharply among older adults. Among older adults, circulatory deaths exhibit the greatest SIIs and RIIs and have the highest standardised mortality rate among men for each education group. For women, the highest standardised mortality rate is in neoplasm deaths, which also has the lowest RI.

Table B.6. Age standardised mortality rates by country, age, education, and cause of death for women

| **Country** | **25-64** | | | | | | | | | | | | **65-89** | | | | | | | | | | | |
| --- | --- | --- | --- | --- | --- | --- | --- | --- | --- | --- | --- | --- | --- | --- | --- | --- | --- | --- | --- | --- | --- | --- | --- | --- |
|  | **Circulatory** | | | **External** | | | **Neoplasms** | | | **Other** | | | **Circulatory** | | | **External** | | | **Neoplasms** | | | **Other** | | |
|  | **L** | **M** | **H** | **L** | **M** | **H** | **L** | **M** | **H** | **L** | **M** | **H** | **L** | **M** | **H** | **L** | **M** | **H** | **L** | **M** | **H** | **L** | **M** | **H** |
| AUS | 35 | 16 | 8 | 32 | 22 | 11 | 91 | 72 | 58 | 77 | 31 | 16 | 408 | 320 | 238 | 51 | 46 | 41 | 635 | 554 | 499 | 663 | 477 | 382 |
| CAN | 49 | 26 | 14 | 36 | 25 | 12 | 132 | 101 | 76 | 112 | 51 | 25 | 560 | 413 | 331 | 61 | 55 | 48 | 891 | 777 | 620 | 755 | 573 | 383 |
| DNK | 44 | 21 | 11 | 31 | 10 | 8 | 132 | 96 | 76 | 131 | 51 | 31 | 523 | 373 | 297 | 47 | 39 | 39 | 945 | 818 | 706 | 1079 | 816 | 625 |
| ESP | 23 | 18 | 8 | 11 | 12 | 5 | 80 | 95 | 54 | 41 | 28 | 11 | 394 | 318 | 222 | 39 | 35 | 26 | 464 | 530 | 393 | 552 | 461 | 325 |
| HUN | 159 | 56 | 23 | 40 | 17 | 11 | 220 | 126 | 85 | 178 | 54 | 25 | 1906 | 1086 | 870 | 87 | 58 | 54 | 1001 | 783 | 790 | 795 | 500 | 421 |
| ITA | 23 | 14 | 10 | 8 | 7 | 6 | 85 | 75 | 67 | 36 | 18 | 13 | 506 | 364 | 324 | 39 | 36 | 33 | 587 | 576 | 551 | 480 | 360 | 323 |
| KOR | 54 | 14 | 7 | 156 | 35 | 15 | 115 | 54 | 45 | 184 | 26 | 12 | 390 | 283 | 232 | 84 | 58 | 52 | 421 | 406 | 390 | 616 | 485 | 418 |
| LTU | 148 | 58 | 25 | 82 | 37 | 20 | 116 | 89 | 66 | 237 | 105 | 54 | 1732 | 1024 | 699 | 128 | 95 | 90 | 619 | 472 | 411 | 955 | 724 | 528 |
| NZL | 42 | 20 | 11 | 21 | 14 | 10 | 119 | 85 | 71 | 71 | 31 | 16 | 666 | 507 | 428 | 52 | 42 | 46 | 732 | 642 | 572 | 760 | 598 | 464 |
| POL | 79 | 47 | 21 | 31 | 16 | 10 | 124 | 116 | 86 | 140 | 62 | 24 | 1365 | 1181 | 748 | 57 | 55 | 43 | 865 | 1069 | 837 | 675 | 657 | 446 |
| SVK | 177 | 43 | 19 | 38 | 15 | 10 | 191 | 99 | 74 | 204 | 57 | 25 | 1861 | 1040 | 633 | 98 | 65 | 49 | 944 | 693 | 561 | 821 | 476 | 309 |
| SWE | 49 | 21 | 10 | 41 | 21 | 11 | 98 | 77 | 58 | 78 | 35 | 16 | 634 | 502 | 315 | 62 | 51 | 42 | 797 | 695 | 552 | 784 | 620 | 414 |
| TUR | 60 | 29 | 20 | 10 | 8 | 6 | 66 | 66 | 56 | 56 | 30 | 19 | 1332 | 683 | 541 | 50 | 36 | 38 | 397 | 458 | 425 | 952 | 630 | 529 |
| USA | 87 | 70 | 29 | 77 | 62 | 23 | 102 | 95 | 64 | 162 | 116 | 46 | 763 | 667 | 468 | 65 | 69 | 59 | 590 | 633 | 525 | 1128 | 975 | 695 |
| **Average** | **74** | **32** | **15** | **44** | **22** | **11** | **119** | **89** | **67** | **122** | **50** | **24** | **932** | **626** | **453** | **66** | **53** | **47** | **706** | **650** | **559** | **787** | **597** | **447** |

Note: L, low education; M, middle education; H, high education. Countries are reported in International Organization for Standardization (ISO) three-letter codes. Education is classified according to the 2011 International Standard Classification of Education (ISCED-2011) into low (lower secondary education and below, ISCED 0-2), medium (upper-secondary, ISCED 3-4), and high education (higher than upper-secondary, ISCED 5-8). Circulatory system diseases (Chapter IX): I00-I99. Neoplasms (Chapter II): C00-D48. External causes (Chapter XX): V01-Y98. Other diseases: All other causes.

Table B.7. Age standardised mortality rates by country, age, sex, education, and cause of death for men

| **Country** | **25-64** | | | | | | | | | | | | **65-89** | | | | | | | | | | | |
| --- | --- | --- | --- | --- | --- | --- | --- | --- | --- | --- | --- | --- | --- | --- | --- | --- | --- | --- | --- | --- | --- | --- | --- | --- |
|  | **Circulatory** | | | **External** | | | **Neoplasms** | | | **Other** | | | **Circulatory** | | | **External** | | | **Neoplasms** | | | **Other** | | |
|  | **L** | **M** | **H** | **L** | **M** | **H** | **L** | **M** | **H** | **L** | **M** | **H** | **L** | **M** | **H** | **L** | **M** | **H** | **L** | **M** | **H** | **L** | **M** | **H** |
| AUS | 85 | 46 | 25 | 114 | 57 | 25 | 134 | 85 | 51 | 123 | 51 | 23 | 758 | 563 | 396 | 101 | 83 | 64 | 1117 | 925 | 666 | 1021 | 679 | 504 |
| CAN | 107 | 75 | 38 | 114 | 69 | 33 | 144 | 105 | 70 | 143 | 77 | 42 | 954 | 739 | 551 | 131 | 107 | 77 | 1338 | 1161 | 786 | 1120 | 855 | 597 |
| DNK | 91 | 51 | 29 | 75 | 38 | 19 | 146 | 99 | 64 | 234 | 110 | 54 | 921 | 795 | 583 | 88 | 74 | 66 | 1322 | 1236 | 950 | 1461 | 1203 | 885 |
| ESP | 67 | 66 | 24 | 40 | 39 | 12 | 132 | 143 | 54 | 88 | 69 | 21 | 778 | 781 | 410 | 94 | 87 | 41 | 1237 | 1333 | 711 | 1078 | 985 | 520 |
| HUN | 499 | 192 | 75 | 179 | 70 | 28 | 424 | 195 | 91 | 391 | 133 | 50 | 3836 | 1903 | 1369 | 290 | 148 | 114 | 2284 | 1338 | 1059 | 1674 | 811 | 596 |
| ITA | 62 | 42 | 29 | 34 | 23 | 16 | 115 | 81 | 62 | 72 | 36 | 23 | 842 | 675 | 589 | 81 | 65 | 62 | 1162 | 983 | 808 | 750 | 580 | 484 |
| KOR | 155 | 49 | 26 | 369 | 109 | 40 | 205 | 93 | 60 | 463 | 90 | 34 | 660 | 554 | 414 | 277 | 177 | 118 | 1202 | 989 | 785 | 1281 | 1000 | 746 |
| LTU | 339 | 209 | 112 | 258 | 160 | 76 | 186 | 146 | 75 | 498 | 282 | 132 | 3084 | 2251 | 1377 | 366 | 257 | 186 | 1520 | 1279 | 814 | 1965 | 1709 | 1002 |
| NZL | 98 | 55 | 36 | 63 | 37 | 24 | 119 | 84 | 63 | 83 | 41 | 25 | 991 | 789 | 607 | 82 | 70 | 80 | 1073 | 945 | 768 | 931 | 745 | 555 |
| POL | 251 | 166 | 67 | 207 | 98 | 35 | 190 | 164 | 82 | 395 | 195 | 65 | 2451 | 2324 | 1155 | 194 | 167 | 77 | 1776 | 2023 | 1140 | 1377 | 1284 | 652 |
| SVK | 535 | 160 | 75 | 234 | 80 | 44 | 386 | 160 | 79 | 560 | 147 | 59 | 4383 | 1895 | 1360 | 358 | 160 | 119 | 2708 | 1428 | 1033 | 2116 | 859 | 605 |
| SWE | 89 | 57 | 28 | 113 | 56 | 25 | 90 | 67 | 50 | 98 | 51 | 29 | 1085 | 849 | 585 | 130 | 107 | 77 | 1036 | 905 | 730 | 938 | 767 | 569 |
| TUR | 136 | 103 | 73 | 47 | 30 | 20 | 142 | 105 | 81 | 97 | 62 | 42 | 1777 | 1339 | 1015 | 103 | 75 | 65 | 1043 | 1007 | 822 | 1431 | 1088 | 828 |
| USA | 173 | 147 | 60 | 205 | 164 | 52 | 133 | 110 | 55 | 229 | 163 | 58 | 1265 | 1185 | 739 | 157 | 161 | 112 | 1004 | 998 | 666 | 1522 | 1336 | 840 |
| **Average** | **192** | **101** | **50** | **147** | **74** | **32** | **182** | **117** | **67** | **248** | **108** | **47** | **1699** | **1189** | **796** | **175** | **124** | **90** | **1416** | **1182** | **838** | **1333** | **993** | **670** |

Note: L, low education; M, middle education; H, high education. Countries are reported in International Organization for Standardization (ISO) three-letter codes. Education is classified according to the 2011 International Standard Classification of Education (ISCED-2011) into low (lower secondary education and below, ISCED 0-2), medium (upper-secondary, ISCED 3-4), and high education (higher than upper-secondary, ISCED 5-8). Circulatory system diseases (Chapter IX): I00-I99. Neoplasms (Chapter II): C00-D48. External causes (Chapter XX): V01-Y98. Other diseases: All other causes

Table B.8. Slope and relative indices of inequality of age-standardised mortality rates by country, age, and cause of death for women

| **Country** | **Females (25-64)** | | | | | | | | **Females (65-89)** | | | | | | | |
| --- | --- | --- | --- | --- | --- | --- | --- | --- | --- | --- | --- | --- | --- | --- | --- | --- |
|  | **Circulatory** | | **External** | | **Neoplasms** | | **Other** | | **Circulatory** | | **External** | | **Neoplasms** | | **Other** | |
|  | **SII** | **RII** | **SII** | **RII** | **SII** | **RII** | **SII** | **RII** | **SII** | **RII** | **SII** | **RII** | **SII** | **RII** | **SII** | **RII** |
| AUS | 34 | 9.633 | 28 | 3.754 | 44 | 1.868 | 75 | 12.22 | 239 | 1.996 | 14 | 1.335 | 201 | 1.414 | 431 | 2.235 |
| CAN | 40 | 6.96 | 31 | 4.837 | 69 | 2.14 | 92 | 11.286 | 325 | 2.19 | 19 | 1.423 | 394 | 1.674 | 538 | 2.6 |
| DNK | 41 | 9.043 | 26 | 16.002 | 72 | 2.219 | 118 | 13.106 | 339 | 2.318 | 14 | 1.426 | 343 | 1.503 | 662 | 2.147 |
| ESP | 25 | 4.677 | 9 | 2.495 | 44 | 1.737 | 49 | 6.552 | 257 | 2.177 | 19 | 1.734 | 23 | 1.051 | 331 | 2.018 |
| HUN | 152 | 17.838 | 33 | 8.273 | 157 | 3.857 | 166 | 25.431 | 1624 | 3.448 | 55 | 2.301 | 373 | 1.559 | 585 | 2.736 |
| ITA | 19 | 3.475 | 3 | 1.467 | 26 | 1.402 | 36 | 4.897 | 306 | 2.059 | 8 | 1.231 | 39 | 1.07 | 261 | 1.886 |
| KOR | 44 | 106.279 | 132 | 226.692 | 64 | 3.766 | 151 | 1803.52 | 245 | 2.122 | 54 | 2.193 | 40 | 1.102 | 301 | 1.749 |
| LTU | 113 | 16.8 | 58 | 8.54 | 57 | 2.047 | 171 | 9.437 | 1377 | 3.497 | 52 | 1.722 | 278 | 1.781 | 556 | 2.096 |
| NZL | 45 | 9.139 | 15 | 2.973 | 69 | 2.248 | 78 | 11.439 | 397 | 2.063 | 12 | 1.305 | 256 | 1.473 | 474 | 2.122 |
| POL | 66 | 4.783 | 21 | 4.917 | 55 | 1.628 | 119 | 9.309 | 624 | 1.638 | 11 | 1.215 | -214 | 0.812 | 174 | 1.294 |
| SVK | 122 | 49.653 | 22 | 7.463 | 99 | 3.497 | 144 | 31.717 | 1646 | 3.719 | 66 | 2.463 | 509 | 1.965 | 688 | 3.386 |
| SWE | 38 | 11.456 | 31 | 6.561 | 48 | 1.999 | 64 | 11.428 | 440 | 2.383 | 27 | 1.701 | 337 | 1.623 | 510 | 2.257 |
| TUR | 71 | 6.152 | 8 | 2.534 | 11 | 1.18 | 62 | 5.223 | 1422 | 4.59 | 27 | 1.862 | -92 | 0.804 | 735 | 2.645 |
| USA | 86 | 4.579 | 81 | 5.095 | 61 | 2.052 | 158 | 5.614 | 414 | 1.931 | 14 | 1.222 | 145 | 1.262 | 596 | 1.915 |
| **Average** | **64.0** | **18.6** | **35.6** | **21.5** | **62.6** | **2.3** | **105.9** | **140.1** | **689.6** | **2.6** | **28.0** | **1.7** | **188.0** | **1.4** | **488.7** | **2.2** |

Note: SII, slope index of inequality; RII, relative index of inequality. Countries are reported in International Organization for Standardization (ISO) three-letter codes. Mortality rates are standardised using the OECD 2010 standard population. Circulatory system diseases (ICD-10 Chapter IX): I00-I99. Neoplasms (ICD-10 Chapter II): C00-D48. External causes (ICD-10 Chapter XX): V01-Y98. Other diseases: All other causes.

Table B.9. Slope and relative indices of inequality of age-standardised mortality rates by country, age, and cause of death for men

| **Country** | **Males (25-64)** | | | | | | | | **Males (65-89)** | | | | | | | |
| --- | --- | --- | --- | --- | --- | --- | --- | --- | --- | --- | --- | --- | --- | --- | --- | --- |
|  | **Circulatory** | | **External** | | **Neoplasms** | | **Other** | | **Circulatory** | | **External** | | **Neoplasms** | | **Other** | |
|  | **SII** | **RII** | **SII** | **RII** | **SII** | **RII** | **SII** | **RII** | **SII** | **RII** | **SII** | **RII** | **SII** | **RII** | **SII** | **RII** |
| AUS | 74 | 5.131 | 110 | 7.042 | 105 | 3.585 | 121 | 10.819 | 483 | 2.212 | 47 | 1.722 | 571 | 1.794 | 729 | 2.635 |
| CAN | 92 | 4.381 | 103 | 6.083 | 95 | 2.766 | 116 | 6.179 | 567 | 2.207 | 78 | 2.14 | 820 | 2.099 | 741 | 2.458 |
| DNK | 81 | 5.113 | 72 | 6.988 | 109 | 3.094 | 232 | 8.312 | 456 | 1.76 | 31 | 1.508 | 492 | 1.487 | 784 | 1.894 |
| ESP | 66 | 3.261 | 42 | 3.694 | 118 | 2.727 | 106 | 5.454 | 489 | 2.044 | 74 | 2.597 | 647 | 1.769 | 801 | 2.426 |
| HUN | 470 | 11.432 | 167 | 10.9 | 375 | 7.096 | 373 | 15.286 | 3323 | 4.913 | 237 | 4.41 | 1647 | 3.21 | 1455 | 5.106 |
| ITA | 47 | 2.651 | 26 | 2.649 | 77 | 2.342 | 75 | 5.102 | 391 | 1.696 | 33 | 1.586 | 481 | 1.584 | 403 | 1.87 |
| KOR | 104 | 36.833 | 278 | 162.855 | 124 | 5.333 | 315 | 2017.65 | 346 | 1.827 | 244 | 3.365 | 609 | 1.8 | 785 | 2.093 |
| LTU | 270 | 3.999 | 220 | 4.334 | 148 | 2.863 | 431 | 5.171 | 2204 | 2.571 | 234 | 2.43 | 905 | 1.99 | 1230 | 2.007 |
| NZL | 88 | 4.66 | 55 | 4.216 | 80 | 2.549 | 81 | 6.332 | 567 | 1.986 | 8 | 1.118 | 434 | 1.57 | 549 | 2.027 |
| POL | 219 | 3.678 | 188 | 6.998 | 143 | 2.306 | 367 | 6.535 | 1273 | 1.671 | 125 | 1.996 | 409 | 1.215 | 729 | 1.701 |
| SVK | 365 | 19.454 | 152 | 13.005 | 269 | 8.366 | 392 | 28.581 | 3065 | 6.068 | 242 | 5.46 | 1741 | 3.833 | 1526 | 7.147 |
| SWE | 75 | 4.163 | 101 | 7.261 | 48 | 2.119 | 78 | 5.501 | 665 | 2.113 | 70 | 1.884 | 403 | 1.544 | 490 | 1.852 |
| TUR | 97 | 2.413 | 44 | 3.569 | 96 | 2.317 | 88 | 3.461 | 1250 | 2.365 | 66 | 2.172 | 289 | 1.344 | 987 | 2.316 |
| USA | 172 | 3.802 | 227 | 4.934 | 112 | 3.197 | 234 | 5.357 | 846 | 2.193 | 82 | 1.731 | 585 | 1.889 | 1018 | 2.32 |
| **Average** | **159** | **7.926** | **127** | **17.466** | **135** | **3.619** | **215** | **152.124** | **1138** | **2.545** | **112** | **2.437** | **717** | **1.938** | **873** | **2.704** |

Note: SII, slope index of inequality; RII, relative index of inequality. Countries are reported in International Organization for Standardization (ISO) three-letter codes. Mortality rates are standardised using the OECD 2010 standard population. Circulatory system diseases (ICD-10 Chapter IX): I00-I99. Neoplasms (ICD-10 Chapter II): C00-D48. External causes (ICD-10 Chapter XX): V01-Y98. Other diseases: All other causes.

### B.7. Robustness analysis

Figure B.7 compares life expectancy estimates at age 25 when either imputing missing education data according to the existing education group proportions or imputing all missing data to low education groups. Positive values indicate a higher life expectancy estimate obtained when imputing all missing education proportionally. For most countries, the difference in imputation method results in minimal changes in life expectancy. However, for the Slovak Republic, the differences in imputation results in larger differences in life expectancy at age 25. Besides, Australia, Hungary, Korea, New Zealand, and Poland also exhibit substantial differences in life expectancy estimates depending on the imputation method.

Figure B.7. Comparison of missing education imputation assumptions


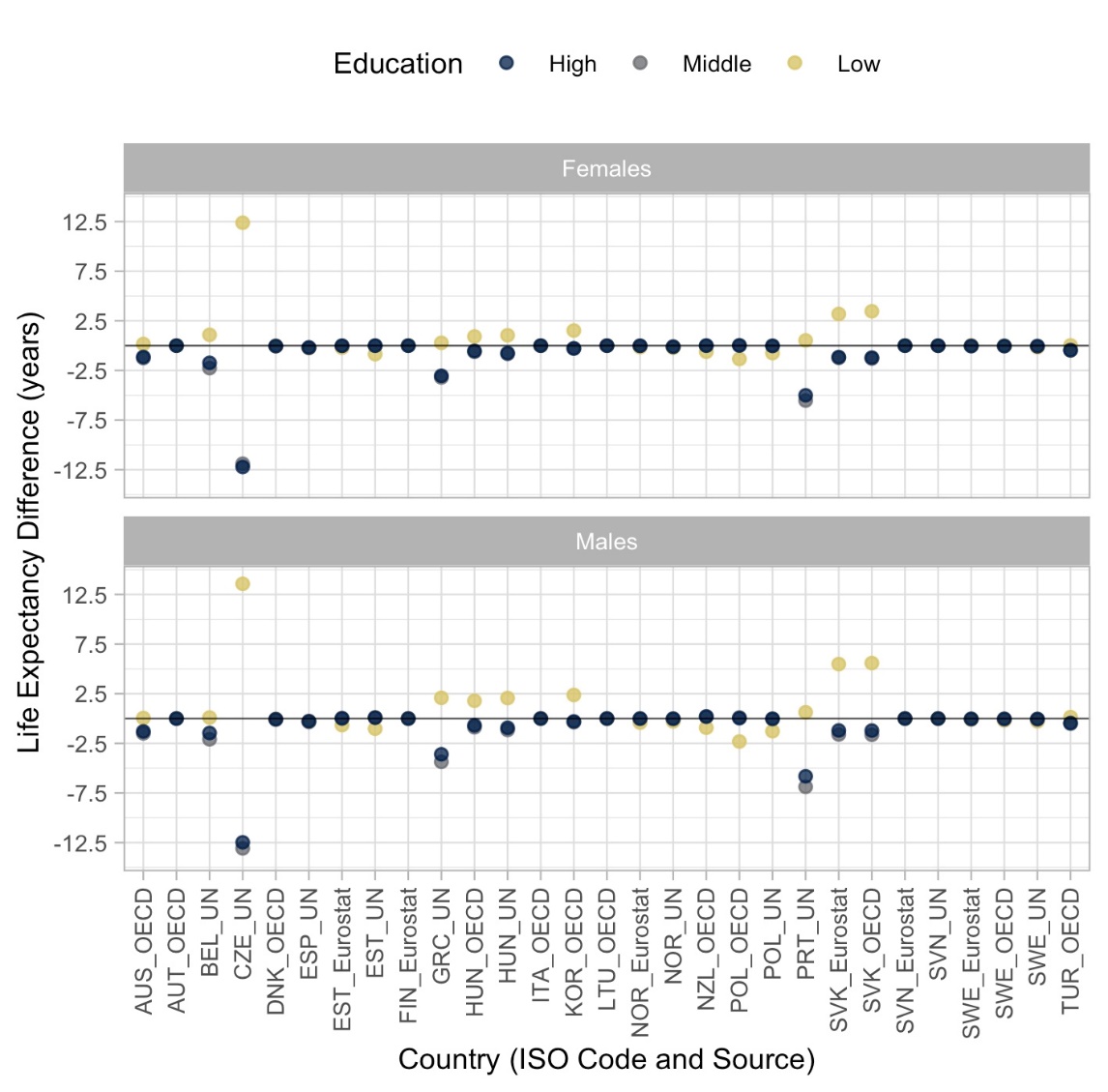


Note: OECD, Organisation for Economic Co-operation and Development. Countries are reported in International Organization for Standardization (ISO) three-letter codes. Positive results indicate higher life expectancy estimates obtained when imputing missing education data to each education group proportionally, compared to imputing all missing education data to the low education group and vice versa.

Finally, to check the robustness of results, the weighted average of life expectancy across low, middle, and high education groups is calculated and compared with life expectancy estimates from the OECD Database, averaged across the same years as used for each country-source (See Table B.10). Due to the data collection methodologies for Japan and Netherlands, educational distribution data are not directly available. To remedy this, the most appropriate data are substituted. For Netherlands, educations from 2011-20 are used for the population aged 25-64 from the OECD Database. The OECD database only contains shares of the population achieving tertiary or higher education level for Japan, so the UN database is used instead: education distribution from 2010 are used for the population aged 25-99. For USA, data on life expectancy at age 40 from 2019 data were not available, so 2018 data are substituted.

The average difference between this paper’s and benchmark life expectancy estimates for women and men is 0.0 and 0.1 years, respectively, at age 40. For life expectancy at age 65, the average difference between our estimates and OECD benchmark is 0.037 years for women and 0.043 years for men. These results indicate slightly higher life expectancy estimates from our analysis than the benchmarks, which may be explained by the methodology of this paper, which considers differential mortality rates by sex and education until age 120. From the primary sources, outliers are Netherlands (1.0 and 0.7 years overestimates for women and men at age 25, respectively) and Lithuania (0.9 years overestimate for men at age 25).

Table B.10. Comparison of analysis and benchmark life expectancy estimates at age 40 by country, source, and sex

|  |  |  |  |  |  |  |  |  |
| --- | --- | --- | --- | --- | --- | --- | --- | --- |
| **Country** | **Source** | **Females** | | |  | **Males** | | |
|  |  | **Analysis** | **Benchmark** | **Difference** |  | **Analysis** | **Benchmark** | **Difference** |
| AUS | OECD | 45.8 | 45.4 | 0.4 |  | 42.2 | 41.8 | 0.4 |
| AUT | OECD | 44.3 | 44.6 | -0.3 |  | 40.3 | 40.5 | -0.2 |
| CAN | OECD | 44.7 | 44.9 | -0.2 |  | 41.2 | 41.3 | -0.1 |
| DNK | OECD | 43.4 | 43.5 | -0.1 |  | 39.7 | 39.8 | -0.1 |
| ESP | OECD | 46.5 | 46.8 | -0.3 |  | 41.4 | 41.5 | -0.1 |
| EST | Eurostat | 43.0 | 43.1 | -0.2 |  | 35.1 | 34.9 | 0.2 |
| FIN | Eurostat | 44.7 | 45.0 | -0.3 |  | 39.7 | 39.8 | -0.2 |
| HUN | OECD | 40.2 | 40.4 | -0.2 |  | 34.0 | 34.0 | 0.0 |
| ITA | OECD | 46.0 | 46.0 | 0.1 |  | 41.9 | 41.6 | 0.3 |
| KOR | OECD | 46.5 | 46.3 | 0.2 |  | 40.6 | 40.5 | 0.1 |
| LTU | OECD | 41.9 | 41.6 | 0.3 |  | 33.7 | 32.8 | 0.9 |
| NLD | OECD | 45.0 | 44.0 | 1.0 |  | 41.8 | 41.1 | 0.7 |
| NOR | Eurostat | 44.8 | 44.8 | 0.0 |  | 41.4 | 41.6 | -0.2 |
| NZL | OECD | 44.4 | 44.4 | 0.0 |  | 41.8 | 41.5 | 0.4 |
| POL | OECD | 42.9 | 42.7 | 0.3 |  | 35.2 | 35.6 | -0.3 |
| PRT | UN | 44.9 | 45.1 | -0.2 |  | 39.4 | 39.2 | 0.2 |
| SVK | OECD | 41.5 | 41.6 | -0.1 |  | 35.3 | 35.3 | 0.0 |
| SVN | Eurostat | 44.3 | 44.5 | -0.2 |  | 39.0 | 39.0 | 0.0 |
| SWE | OECD | 44.8 | 44.9 | -0.1 |  | 41.7 | 41.8 | 0.0 |
| TUR | OECD | 42.5 | 42.4 | 0.1 |  | 37.9 | 37.6 | 0.4 |
| USA | OECD | 42.8 | 42.7 | 0.1 |  | 38.8 | 38.7 | 0.1 |
| **Average** | | **44.0** | **44.0** | **0.0** |  | **39.1** | **39.0** | **0.1** |

Note: OECD, Organisation for Economic Co-operation and Development. Countries are reported in International Organization for Standardization (ISO) three-letter codes.
